# Targeting the HSP60/p53 Axis with Extracellular Vesicle-Delivered siRNA Reprograms Glycolysis in Prostate Cancer

**DOI:** 10.7150/ijbs.120760

**Published:** 2026-01-01

**Authors:** Meng-Yao Xu, Sheng Ma, Si-Yang Ma, Chen-Qian Liu, Jian-Xuan Sun, Ye An, Jin-Zhou Xu, Si-Han Zhang, Na Zeng, Xing-Yu Zhong, Xiao-Hua Zhu, Shao-Gang Wang, Qi-Dong Xia

**Affiliations:** 1 Department of Urology, Tongji Hospital, Tongji Medical College, Huazhong University of Science and Technology, No. 1095 Jiefang Avenue, 430030, Wuhan, China.; 2 Department of Nuclear Medicine, Tongji Hospital, Tongji Medical College, Huazhong University of Science and Technology, No. 1095 Jiefang Avenue, 430030, Wuhan, China.; 3 Laboratory of Signaling and Gene Regulation, Cecil H. and Ida Green Center for Reproductive Biology Sciences, University of Texas Southwestern Medical Center, Dallas, TX 75390, USA.

**Keywords:** prostate cancer, HSP60, p53, glycolysis, extracellular vesicles

## Abstract

Prostate cancer (PCa), a most prevalent urologic malignancy in men, remains a therapeutic challenge due to limited targeted strategies. This study investigates heat shock protein 60 (HSP60) (*HSPD1*-encoded), employing multi-dimensional approaches to decipher its oncogenic role and develop siRNA-loaded extracellular vesicles (siRNA@EVs) for PCa targeted therapy. Bioinformatics screening identified *HSPD1* overexpression in PCa, which was validated via qPCR/Western blot in clinical tissues and cell lines. Metabolomic-transcriptomic integration and molecular biology experiments revealed HSP60-mediated glycolytic reprogramming. EVs were harvested from UV-irradiated PCa cells via high-speed centrifugation. siRNA@EVs were constructed via electroporation and evaluated *in vitro* (glycolysis phenotyping: glucose consumption, lactate/pyruvate production, hexokinase activity, and ATP production) and *in vivo* using xenograft models. Data were analyzed using R 4.3.1 and GraphPad Prism 9.0 (two-tailed t-test,* P* < 0.05). Multiple bioinformatics analyses (DepMap/TCGA/HPA) confirmed that HSP60 is specifically overexpressed and associated with advanced PCa progression and poor prognosis. *HSPD1* knockdown and pharmacological HSP60 inhibition suppressed proliferation, metastasis, and subcutaneous tumor growth, while overexpression exacerbated oncogenicity. Multi-omics integration revealed HSP60 enhances glycolysis via p53 suppression, driving metabolic reprogramming. siRNA@EVs achieved significant *HSPD1* silencing, effectively inhibiting the proliferation and metastasis of PCa cells, and blocking xenografts tumor growth in nude mice with safety. siRNA@EVs targeting *HSPD1* demonstrate precision therapeutic potential with robust efficacy and safety, offering a novel approach for targeted therapy in PCa.

## Introduction

Prostate cancer (PCa), ranking among the most prevalent malignancies in males worldwide, is characterized by high morbidity and mortality rates. Recent cancer statistics reveal that PCa accounts for 29% of all male malignancies in the United States, maintaining the highest incidence rate and second-leading cancer-related mortality among men 1. In China, it has emerged as the predominant urogenital malignancy with persistently rising incidence rates 2. Disease progression from localized to locally advanced and ultimately metastatic PCa frequently leads to acquired resistance to conventional androgen deprivation therapy (ADT) and anti-androgen agents (e.g., enzalutamide, abiraterone acetate), culminating in castration-resistant prostate cancer (CRPC) 3. The high recurrence rate and dismal survival outcomes associated with CRPC present a major clinical challenge 4.

The rapid advancement of molecular biology and precision oncology has positioned targeted therapy as a pivotal research direction in cancer treatment, offering novel therapeutic alternatives for advanced PCa refractory to hormonal interventions 5, 6. Nevertheless, current therapeutic targets face substantial limitations, including inadequate tissue specificity, off-target toxicity, and inevitable drug resistance 7, 8. Consequently, the identification of novel molecular targets, elucidation of their mechanistic roles in prostate carcinogenesis and progression, and development of precision diagnostic-therapeutic strategies constitute urgent priorities in PCa research.

Heat shock protein 60 (HSP60), a highly conserved chaperonin in the heat shock protein family encoded by the *HSPD1* gene, is primarily localized to the mitochondrial matrix where it participates in fundamental biological processes including protein folding, assembly, and stabilization 9. Emerging evidence indicates that HSP60 not only maintains cellular homeostasis but also plays pivotal regulatory roles in tumorigenesis and progression 10. Intriguingly, HSP60 overexpression has been consistently observed across multiple malignancies, including but not limited to bladder, pancreatic, and breast carcinomas 11-13, suggesting its cancer-specific functional implications. Furthermore, HSP60 occupies a central position in tumor metabolism, orchestrating multidimensional metabolic adaptation through its regulation of critical pathways encompassing glycolysis, oxidative phosphorylation, lipid metabolism, and oxidative stress in cancer cells 14.

Metabolic reprogramming, a hallmark of malignant tumors, features aberrantly activated glycolysis as a critical metabolic adaptation enabling cancer cells to thrive in hostile microenvironments and sustain rapid proliferation 15, 16. The Warburg effect, first described in tumor biology, reveals cancer cells' preferential reliance on glycolytic pathways for energy production even under normoxic conditions 16. This metabolic rewiring not only provides energetic substrates through dysregulated glucose uptake and fermentation, but more fundamentally, sustains biosynthetic precursors for nucleic acids, lipids, and amino acids—thereby fueling uncontrolled proliferation and metastatic potential 17. In PCa evolution, glycolytic activation is orchestrated through multilayered molecular networks. Beyond classical metabolic enzymes, emerging evidence identifies molecular chaperones with non-classical regulatory roles in glycolytic control, among which the metabolic governance of HSP60 warrants particular attention 18. Contemporary studies further reveal HSP60's functional duality: while executing intracellular chaperone activities, it also exhibits extracellular secretion capacity with demonstrated immunomodulatory and signal transduction properties 19. Notably, tumor-associated HSP60 overexpression correlates strongly with enhanced proliferative capacity, apoptosis resistance, and metastatic competence in malignancies 20, 21. However, the mechanistic underpinnings of HSP60-mediated metabolic reprogramming in PCa progression remain incompletely resolved, particularly its regulatory crosstalk with glycolytic circuitry—a critical knowledge gap demanding systematic investigation.

The rapid evolution of precision medicine has positioned HSP60-targeted therapeutic strategies as promising frontiers in PCa management. Nevertheless, the clinical translation of this paradigm faces a critical challenge: optimizing the delivery efficiency and tumor specificity of therapeutic agents. Extracellular vesicles (EVs), naturally occurring nanoscale particles with lipid bilayer membranes, exhibit inherent advantages for molecular delivery, including protection of cargo from enzymatic degradation, reduced immune clearance, and intrinsic targeting capabilities through surface biomarkers 22, 23. Electroporation-mediated loading of *HSPD1*-targeting siRNA (si-*HSPD1*) into EVs enables construction of potent RNA interference systems for precise gene silencing. Notably, EVs demonstrate preferential tumor tropism, as malignant cell-derived EVs are preferentially internalized by tumor cells through homologous uptake mechanisms, thereby enhancing therapeutic delivery precision 24. This biological synergy between EVs biogenesis and tumor pathophysiology provides a rational strategy for developing HSP60-targeted therapies. Specifically, EV-mediated delivery of si-*HSPD1* capitalizes on both the molecular specificity of RNA interference and the tumor-targeting properties of endogenous vesicle systems 25, establishing a novel theoretical framework for precision intervention in PCa.

Guided by these insights, this investigation established a translational research paradigm to address HSP60-driven PCa progression through three synergistic dimensions. Firstly, we rigorously validated HSP60 as a potential therapeutic target demonstrating tumor-specific overexpression in PCa. Secondly, we employed multi-omics approaches to elucidate its mechanism of promoting glycolytic reprogramming in PCa. Thirdly, we innovatively constructed an siRNA delivery system targeting HSP60--therapeutic EVs loaded with siRNA (siRNA@EVs). This integrated approach establishes a translational continuum from mechanistic elucidation to therapeutic implementation, providing both mechanistic clarity for cancer metabolism and a clinically viable blueprint for precision therapy.

## Methods

### Bioinformatics analysis

By integrating functional genomic annotations with systematic phenotypic screening strategies, this study first leveraged genome-wide CRISPR-Cas9 loss-of-function screening data from the Q2 2024 release of the Cancer Dependency Map (DepMap; https://depmap.org/portal/), focusing on PCa cell lines (PC-3, DU145, 22Rv1, C4-2, and LNCaP). Through a multi-step bioinformatic prioritization pipeline, we identified candidate dependency genes critically regulating PCa cell proliferation and survival.

Paired RNA sequencing (RNA-Seq) data from tumor tissues and matched adjacent normal tissues were retrieved from The Cancer Genome Atlas Prostate Adenocarcinoma (TCGA-PRAD) database (https://portal.gdc.cancer.gov/). Differential expression analysis was performed using the DESeq2 package (v1.38.3) in R, with subsequent identification of mRNAs significantly upregulated in PCa tissues.

The expression data of candidate proteins across tissues were retrieved from the Human Protein Atlas (HPA) database (https://www.proteinatlas.org/) for comprehensive validation, including: 1) Expression profiles in normal prostate/glandular cells; 2) Expression levels in prostate tumor tissues; 3) Subcellular localization patterns. Through these criteria, we identified specific target proteins demonstrating elevated expression in prostate carcinoma while maintaining minimal expression in normal prostate tissues.

### Human PCa tissues

The Tongji PCa cohort includes 96 PCa patients from Tongji Hospital, and the tissues were obtained from the resected PCa tissue and adjacent tissues of these patients during surgery. To ensure the adjacent "non-tumorous" tissues were free of cancerous cells, all tissue samples were meticulously evaluated by two experienced surgeons and pathologists. The adjacent tissues were collected at a minimum distance of 2 cm from the macroscopic tumor margin. The clinical information of the patient was shown in Supplementary Table 1.

### Cell culture

The human normal prostate epithelial cell line RWPE-1 and PCa cell lines (22Rv1, C4-2, DU-145, LNCaP, PC-3) were purchased from ATCC (American Type Culture Collection). RWPE-1 cells were cultured in RWPE-1-specific complete medium; 22Rv1, C4-2, and LNCaP cell lines in RPMI-1640 medium; DU-145 cells in high-glucose DMEM; and PC-3 cells in Ham's F-12K medium. All basal media formulations were supplemented with 10% fetal bovine serum (FBS) and 1% penicillin/streptomycin solution. Cells were incubated at 37 °C in a 5% CO₂-humidified atmosphere.

### Total RNA extraction, reverse transcription, quantitative real-time polymerase chain reaction (qPCR)

Total RNA was extracted from prostatic epithelial and cancer cells using Total RNA Reagent (Trizol, ABclonal, China) according to the manufacturer's protocol. Reverse transcription was performed with Hifair III RT Buffer (Yeasen, China) for cDNA synthesis. QPCR analysis was carried out using Hieff qPCR SYBR Green Master Mix (Low Rox Plus) kit (Yeasen, China) to determine mRNA levels. Relative gene expression was calculated by the 2^-△△ Ct^ method using GAPDH as the endogenous control. Primer sequences are detailed in Supplementary Table 2.

### Protein extraction and western blot

Total protein extraction was performed using ice-cold RIPA lysis buffer (Solarbio, China) supplemented with protease and phosphatase inhibitors. Protein concentration was determined by BCA protein quantification kit (BOSTER, China). Protein samples were mixed with loading buffer (Seven, China) and heat-denatured prior to electrophoresis.

Equal protein quantities from each sample were separated by SDS-polyacrylamide gel electrophoresis (SDS-PAGE) using PAGE Gel Fast Preparation Kit (EpiZyme, China), followed by electrophoretic transfer to PVDF membranes. Nonspecific proteins on the membrane were blocked with fast blocking buffer, and then the membranes were incubated in primary antibody diluent at 4 °C overnight. After TBST washing, membranes were incubated with HRP-conjugated secondary antibodies (Abclonal, Wuhan, China) for 1 h at room temperature. All antibodies used in this study are shown in Supplementary Table 3. Protein bands were wetted with ECL chemiluminescent substrate (BOSTER, China) and subsequently visualized using chemiluminescence imaging system (Bio-Rad, USA). Quantitative analysis was performed through band intensity normalization using Image J software.

### Plasmids construction, lentivirus transfection, and siRNA transfection

Lentiviruses with knockdown and overexpression of *HSPD1* gene were purchased from Genomeditech (Shanghai, China). Cy3-labeled siRNA oligonucleotides targeting *HSPD1* were obtained from GenTarget (Wuhan, China). The sequences of all shRNA and siRNA oligonucleotides are provided in Supplementary Table 4.

22Rv1 and C4-2 cells were transfected with lentivirus at a multiplicity of infection (MOI) of 5 supplemented with Polybrene (6 μg/mL) following the manufacturer's protocol. The culture medium was replaced 12~24 hours post-transfection, followed by selection in complete medium containing puromycin (2 μg/mL) for 7 days to establish stable transfected cell lines. For siRNA transfection, transfection complexes were prepared by mixing optimized serum-free medium, siRNA oligonucleotides, and RNA Rocket transfection reagent according to the manufacturer's protocol. The transfection reagent was used to transfect cells, and gene expression detection was performed 48h later.

### Cell Counting Kit-8 (CCK-8) assay

The cell activity at different intervention times were determined using Cell Counting Kit-8 (CCK-8) (MCE, USA). Inoculate untreated 22Rv1 and C4-2 cells (100 μL, 1 × 10^4^ cells/well) onto a 96 well plate and incubate at 5% CO_2_ and 37 °C for 24 hours. Afterwards, cells were intervened with different drugs. After intervention at different times, 10 μL of CCK8 solution mixed with 90 μl of serum-free medium was added to each well of the plate and incubated for 2 hours (5% CO_2_, 37 °C). Use microplate reader (Model 680, BIO-RAD, USA) to measure the absorbance at 450 nm.

### EdU assay

Inoculate cells into a 24 well plate (8×10^4^ cells/well) and culture for 24 hours under standard conditions (5% CO₂, 37 °C). Cell proliferation was assessed using the EdU Cell Proliferation Assay Kit (Beyotime Biotechnology, Shanghai, China) following the manufacturer's protocol. Fluorescence microscopy (Bio-Rad, CA, USA) was employed to capture blue channel images for Hoechst-stained nuclei and red channel images for EdU-positive cells. The proliferation rate was quantified as the percentage of EdU-positive cells relative to total Hoechst-stained nuclei.

### Colony formation assay

Cells subjected to distinct genetic or pharmacological perturbations were seeded into 6-well plates at a density of 800 cells/well in complete growth medium. Following 14 days of culture under standard conditions, colonies containing ≥ 50 cells were quantified through crystal violet staining. Comparative analysis demonstrated significant differences in clonogenic capacity between experimental cohorts (*P* < 0.05, two-tailed Student's t-test).

### Wound healing assay

The inserts were placed in 6-well plates, and pretreated cells were seeded into the central chamber of each insert. Upon reaching full confluence across the insert surface, the inserts were carefully removed with sterile forceps to generate a uniform scratch wound. Following three PBS washes to eliminate mechanically dislodged cells, serum-free RPMI 1640 medium was added, and cells were maintained under standard culture conditions for 72 hours. Cell migration into the denuded area was monitored using an optical microscope (10x) (Olympus, Japan), with photomicrographs acquired at 24-hour intervals. The wound migration was quantified by measuring the reduction in scratch area using ImageJ software.

### Transwell assay

The migratory and invasive capacities of 22Rv1 cells were evaluated using Corning Transwell inserts (8.0 μm pore polycarbonate membranes). Briefly, 22Rv1 cells (1 x 10^5^ cells/ chamber) were seeded into the upper chamber in serum-free medium, while the lower chamber contained complete medium supplemented with 20% FBS as a chemoattractant. Following a 48-hour incubation, cells that had traversed the membrane were fixed with 4% paraformaldehyde, stained with crystal violet (Beyotime), and quantified under a computerized microscope.

### Apoptosis flow cytometry assay

Apoptotic cells were quantified using an Annexin V-APC/PI dual-staining apoptosis detection kit (KGA, Nanjing, China) according to the manufacturer's protocol. Briefly, stained cells were analyzed by flow cytometry, and the percentages of early apoptotic (Annexin V-APC⁺/PI⁻), late apoptotic (Annexin V-APC⁺/PI⁺), necrotic (Annexin V-APC⁻/PI⁺), and viable (Annexin V-APC⁻/PI⁻) populations were determined using FlowJo software.

### Immunohistochemistry and H&E analysis

For IHC, tissue sections were incubated with corresponding antibodies, followed by incubation with HRP enzyme labeled secondary antibodies. Finally, DAB was used for color visualization and images were observed and captured under a fluorescence microscope. For H&E assay, tissue sections are dewaxed, rehydrated, stained with hematoxylin eosin, and observed and captured under a fluorescence microscope.

### Untargeted metabolomic analysis

Untargeted metabolomics was conducted on *HSPD1*-knockdown 22Rv1 and C4-2 PCa cell lines (vs. isogenic controls) to identify *HSPD1*-dependent metabolic alterations. Experimental workflows, performed by Wuhan Maiwei Metabolomics Biotechnology Co., Ltd., included cell harvesting at 80% confluence, metabolite extraction using standardized solvent systems, and LC-MS analysis with dual-polarity electrospray ionization. Acquired data were processed, followed by multivariate statistical modeling, pathway enrichment analysis, and hierarchical clustering to delineate reprogrammed metabolic networks.

### Transcriptome Sequencing

RNA sequencing (RNA-Seq) was performed on *HSPD1*-knockdown 22Rv1 and C4-2 PCa cell lines alongside their isogenic controls. All experimental procedures, executed by Shanghai OE Biotech Co., Ltd., encompassed RNA isolation, quality verification, cDNA library construction, paired-end sequencing, and subsequent bioinformatic processing, including read alignment, differential gene expression analysis, and pathway enrichment studies.

### Glycolysis-related phenotype assay

Glucose consumption was measured using Glucose Kit (glucose oxidase method) (Nanjing Jiancheng Bioengineering Institute, Nanjing, China). Lactic acid content determination was measured using CheKine™ Micro Lactate Assay Kit (Abbkine, USA). Pyruvate content determination was measured using CheKine™ Micro Pyruvate Acid (PA) Assay Kit (Abbkine, USA). Hexokinase (HK) activity assay was measured using CheKine™ Micro Hexokinase (HK) Activity Assay Kit (Abbkine, USA). ATP content determination was measured using CheKine™ Micro ATP Content Assay Kit (Abbkine, USA). The above operations were carried out according to the manufacturer's protocol.

### Extracellular acidification rate (ECAR) analysis

Cells were seeded in XF96 plates (22Rv1: 2×10⁴; C4-2: 1.5×10⁴ cells/well) and cultured for 24 h. Prior to assay, culture medium was replaced with Seahorse XF RPMI (pH 7.4) containing 2 mM glutamine. ECAR was monitored using the Glycolysis Stress Test Kit (Agilent) with sequential injections of 11 mM glucose, 1.5 μM oligomycin, and 50 mM 2-deoxyglucose. The experimental results were analyzed using Wave Program 2.6.0 (Seahorse Bioscience).

### Isolation of PCa cell-derived extracellular vesicles (EVs)

EVs were isolated from PCa cells cultured to 70-80% confluence. Following three gentle PBS washes to remove pre-existing EVs, serum-free medium was added into the culture dish. UV-irradiated (1 hour) prior to 24-hour incubation under standard culture conditions. Cell supernatants were sequentially cleared by centrifugation (1 500 ×g, 10 min; 5 000 ×g, 15 min) to remove cellular debris. EV-enriched pellets were obtained through high-speed centrifugation (18 000 ×g, 60 min, 4 °C), washed with PBS, and resuspended in sterile PBS for storage at -80 °C.

### Characterization of EVs

EVs were comprehensively characterized through multimodal analytical approaches. Morphological evaluation via transmission electron microscopy (TEM) revealed characteristic cup-shaped vesicle structures. Nanoparticle Tracking Analysis (NTA) quantified particle size distribution, while zeta potential measurements (ZetaView PMX-120, Particle Metrix) assessed surface charge stability. Western blotting confirmed enrichment of EV-specific markers CD63, CD9, and ALIX, with calnexin serving as a negative control to exclude endoplasmic reticulum contamination.

### Electroporation of siRNA-loaded extracellular vesicles (EVs@si-*HSPD1*)

EVs were electroporated with *HSPD1*-targeting siRNA using optimized electroporation parameters. Briefly, EVs (1×10¹² particles/mL in electroporation buffer) were mixed with 20 μM siRNA (RNase-free) under gentle agitation. Pre-chilled samples (4 °C, 10 min) were loaded into electroporation cuvettes and subjected to three sequential pulses (580 V, 120 ms pulse duration) with 1-minute recovery intervals. Post-electroporation, samples were incubated at 37 °C for 1 hour to facilitate membrane reconstitution, followed by two rounds of PBS buffer exchange. Final EV@si-*HSPD1* suspensions were stored in sterile PBS at -80 °C until functional validation.

### Detection of EVs uptake by PCa cells

PKH67-labeled EVs (30 μg) were incubated with PCa cells seeded at 10000 cells/well in confocal-compatible dishes for 3-6 hours under standard culture conditions, with unlabeled EVs serving as autofluorescence controls. Post-incubation, cells underwent three PBS washes, fixation (4% paraformaldehyde, 20 min), and permeabilization (0.2% Triton X-100/5% BSA, 90 min). Nuclei were counterstained with DAPI (1:1,000, 10 min), and samples were mounted with anti-fluorescence quenching reagent for imaging via immunofluorescence microscopy.

### Organoid models

Primary prostate cancer tissues were acquired through surgical resection from consenting patients and subsequently processed for organoid derivation. Following aseptic dissection, tissue specimens were mechanically minced and enzymatically disaggregated in a customized digestion cocktail comprising TrypLE (Gibco) supplemented with 10 μM Y-27632 (ROCK inhibitor). To enrich luminal epithelial populations, single-cell suspensions were subjected to fluorescence-activated cell sorting (FACS; BD Aria II) using established surface markers. Cell isolation protocols were performed as previously described 26. Briefly, FACS-purified cells were pelleted by centrifugation (300 ×g, 5 min) and subsequently suspended in growth factor-reduced Matrigel for 3D culture initiation.

For quantitative organoid formation assessment, a standardized density of 2×10³ cells/well was seeded in 30 μL Matrigel domes. Cultures were incubated in a humidified 37 °C/5% CO₂ chamber with medium changes every 48 hours.

### PCa xenograft models in BALB/c nude mice

Male BALB/c nude mice aged 8 weeks were purchased from Hunan SJA Laboratory Animal Co., Ltd. We kept the mice in the SPF experimental animal center and let them adapt to the environment for one week. To observe the effect of *HSPD1* on tumor growth *in vivo*, nude mice were randomized into sh-*HSPD1* and sh-NC control groups (n = 5/group). Subcutaneous xenografts were established via axillary injection of 3×10⁶ 22Rv1 or C4-2 cells suspended in 100 μL PBS. After the subcutaneous transplant tumor grew to a volume of 50-80 mm^3^, we observed and recorded the volume of the transplanted tumor until the maximum diameter of the tumor approached 20 mm. For siRNA@EVs therapeutic evaluation, mice bearing 50-80 mm³ tumors received three tail vein injections (3-day intervals) of siRNA@EVs, EVs, siRNA, EVs+siRNA or PBS. When the maximum diameter of the subcutaneous transplant tumor approached 20 mm, CO_2_ was used to euthanize nude mice. We then removed the subcutaneous transplant tumor, measured the volume and mass, and fixed the tumor tissue with formaldehyde for subsequent experiments.

We calculated tumor size using the following formula: Volume=1/2 x (Length x Width^2^).

### Statistical analysis

The mean ± SD represents the data results of at least three independent experiments. All statistical analyses were conducted using GraphPad Prism (V.8.0.3, San Diego, USA). Kaplan-Meier survival analysis was used to evaluate the association between gene expression and the probability of survival in multiple tumors, and we used logarithmic rank tests to determine *P* values, HRs, and 95% confidence intervals (CI). We evaluated statistical significance through bilateral unpaired Student's t-test or one-way ANOVA, followed by Dunnett's post-hoc test. Spearman correlation analysis was used to detect gene co expression. For functional experiments, the legend in the relevant figure represents the specific statistical tests used. *P* value < 0.05 was considered statistically significant.

## Results

### HSP60 is specifically overexpressed in PCa and is associated with PCa progression and poor prognosis

**Figure 1:**
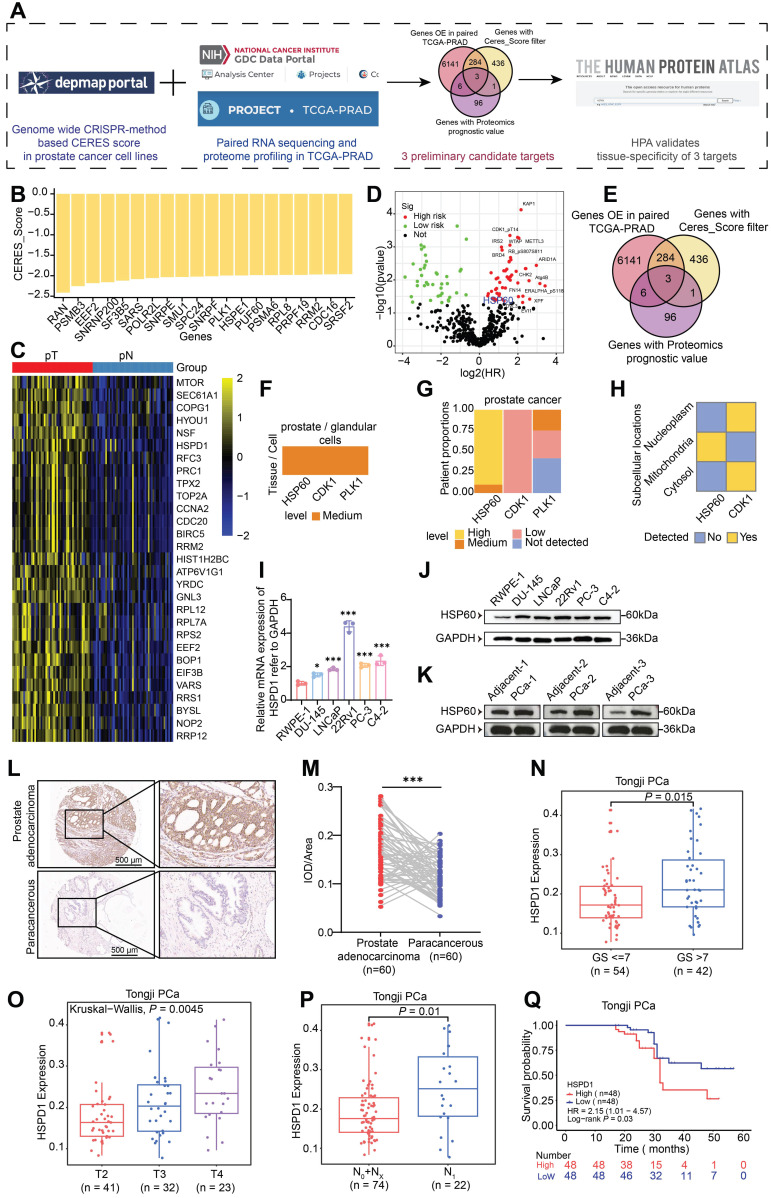
** HSP60 is highly expressed in PCa and is associated with PCa progression and poor prognosis. (A)** Screening flowchart for target molecules. **(B)** CRISPR screening data from DepMap database validating DNA-level proliferation-associated genes in PCa cells. **(C)** Transcriptomic analysis of TCGA-PRAD paired tissues identifying mRNA overexpression in PCa. **(D)** Proteomic profiling using TCGA database to identify PCa risk factors at protein level. **(E)** Venn diagram showing overlapping genes/proteins across three databases.** (F)** Expression patterns of three proteins in prostate glandular cells. **(G)** Expression and proportions of three proteins in PCa patients. **(H)** Subcellular localization of HSP60 and CDK1. **(I)** qPCR analysis of *HSPD1* mRNA levels in RWPE-1 versus DU145, LNCaP, 22Rv1, PC-3, and C4-2 cells. **(J)** Western blot detection of HSP60 protein in RWPE-1 versus DU145, LNCaP, 22Rv1, PC-3, and C4-2 cells. **(K)** Western blot detection of HSP60 protein in cancerous tissues versus adjacent normal tissues from prostate cancer patients. **(L)** Representative IHC images of HSP60 in prostate adenocarcinoma and paracancerous tissues.** (M)** Semiquantitative scoring of HSP60-positive areas in tumor versus paracancerous tissues. **(N-Q)** Association between *HSPD1* expression and GS** (N)**, T-stage **(O)**, lymph node metastasis **(P)**, or biochemical recurrence **(Q)** in Tongji PCa cohort. Statistical analysis is performed using two-sided t-test **(I), (N), (O), (P), (Q)** and a paired sample t-test for panel **(M)**; Means ± SD, *P < 0.05; **P < 0.01; ***P < 0.001;

To systematically identify molecular targets with PCa-specific overexpression, this study conducted integrative analyses of multiple public databases **(Fig. 1A)**. Initially, Clustered Regularly Interspaced Short Palindromic Repeats (CRISPR) functional genomics screening using Dependency Map portal (DepMap) revealed 724 lineage-specific essential genes in PCa cell lines, demonstrating a significant inverse correlation between Gene Effect Score (CERES) and baseline expression levels (*P* < 0.05). **Fig. 1B** highlights the 20 genes with the lowest mean CERES scores across all PCa cell lines, suggesting their knockdown may substantially impair tumor cell survival. Subsequent transcriptomic analysis of TCGA-PRAD datasets identified > 6000 mRNAs significantly upregulated in prostate adenocarcinoma tissues, with *HSPD1* (encoding HSP60) showing particularly pronounced overexpression **(Fig. 1C)**. To validate clinical relevance, we systematically analyzed TCGA PCa proteomics data, identifying 106 hazard-associated proteins significantly correlated with patient prognosis** (Fig. 1D)**. Through this multi-omics integrative approach, three candidate targets emerged: HSP60, CDK1, and PLK1** (Fig. 1E)**. These findings suggest these molecules may orchestrate PCa progression through multi-layered molecular regulatory networks, warranting further mechanistic investigation. Tissue-specific expression and subcellular compartmentalization of candidates were validated via the HPA. While HSP60, CDK1 and PLK1 showed medium expression in normal prostatic glandular cells, only HSP60 demonstrated marked overexpression in PCa patients and exclusive mitochondrial localization **(Fig. 1F-H, Fig. S1B)**. IHC analysis confirmed cancer-specific enrichment of strong HSP60 positivity compared to benign tissues, a pattern absents for CDK1 and PLK1 **(Fig. S1A)**. This dual specificity—tissue-restricted overexpression and organelle-confined localization—positions HSP60 as a mechanistically distinct therapeutic target.

We performed multi-dimensional HSP60 profiling across cell line and clinical tissue specimens. At the cellular level, comparative analysis of 5 PCa cell lines versus normal prostate epithelial cells revealed significant upregulation of both *HSPD1* mRNA and HSP60 protein (*P* < 0.05), with 22Rv1 and C4-2 cells showing the most pronounced elevation** (Fig. 1I-J, Fig. S1C)**. In patient tissues, western blot analysis consistently demonstrated a significant upregulation of HSP60 protein in PCa tissues compared to their matched adjacent normal tissues** (Fig. 1K, Fig. S1D).** And HSP60 exhibited robust cytoplasmic positivity in malignant tissues **(Fig. 1L-M)**, contrasting sharply with its minimal detection in paracancerous tissues. In addition, compared with tissues from patients with benign prostatic hyperplasia (BPH), HSP60 also showed strong positive expression in PCa tissues **(Fig. S1E)**. Collectively, these findings suggest that HSP60 is specifically overexpressed in PCa.

To investigate HSP60's clinicopathological relevance in PCa, we integrated 96 institutional patients' data with paired tumor/adjacent tissue profiles. Strikingly, *HSPD1* expression positively correlated with the malignancy of PCa, showing elevated *HSPD1* levels in Gleason score (GS) > 7 tumors (*P* = 0.015) **(Fig. 1N)**. *HSPD1* expression increased incrementally with advancing T-stage (*P* = 0.0045) **(Fig. 1O)**. And N1 patients exhibited higher *HSPD1* expression than N0/NX cohorts (*P* = 0.01) **(Fig. 1P)**. Critically, *HSPD1*-high patients exhibited shorter biochemical recurrence-free survival (HR = 2.15, 95% CI: 1.01-4.57; *P* = 0.03) **(Fig. 1Q)**. In addition, this study also conducted a systematic analysis of clinical signs on TCGA-PRAD data and confirmed concordant findings **(Fig. S1F-I)**. These findings position *HSPD1* as a mechanistically relevant prognostic biomarker, with its expression levels directly correlating with PCa aggressivity and adverse clinical outcomes.

### HSP60 promotes proliferation and metastasis of PCa cells

To explore HSP60's functional role in PCa, we employed Nonactin—a specific HSP60 inhibitor—in pharmacological perturbation assays 27. Colony formation assays demonstrated marked suppression of clonogenic capacity in Nonactin-treated versus control groups **(Fig. S2A)**. CCK-8 assays demonstrated time-dependent cytotoxicity, with 10 μM Nonactin reducing 22Rv1 viability by 25% (24 h), 33% (48 h), and 40% (72 h), and C4-2 viability by 23%, 27%, and 35%, respectively (*P* < 0.001)** (Fig. S2B)**. EdU assay corroborated these findings, showing significantly diminished proliferative fractions in Nonactin-treated cells versus control groups control **(Fig. S2C)**. To functionally validate Nonactin's impact on metastatic competence, we conducted wound healing and Transwell migration/invasion assays. Wound healing analysis revealed Nonactin-treated cells exhibited significantly larger residual wound areas compared to control group at matched timepoints, indicating impaired migratory capacity **(Fig. S2D)**. The results of Transwell assays showed that compared with the control group, the number of cells migrating and invading through the porous membrane in the Nonactin group was significantly reduced **(Fig. S2E)**. These collective findings demonstrate Nonactin potently suppresses PCa cell motility and matrix-infiltrative potential.

**Figure 2:**
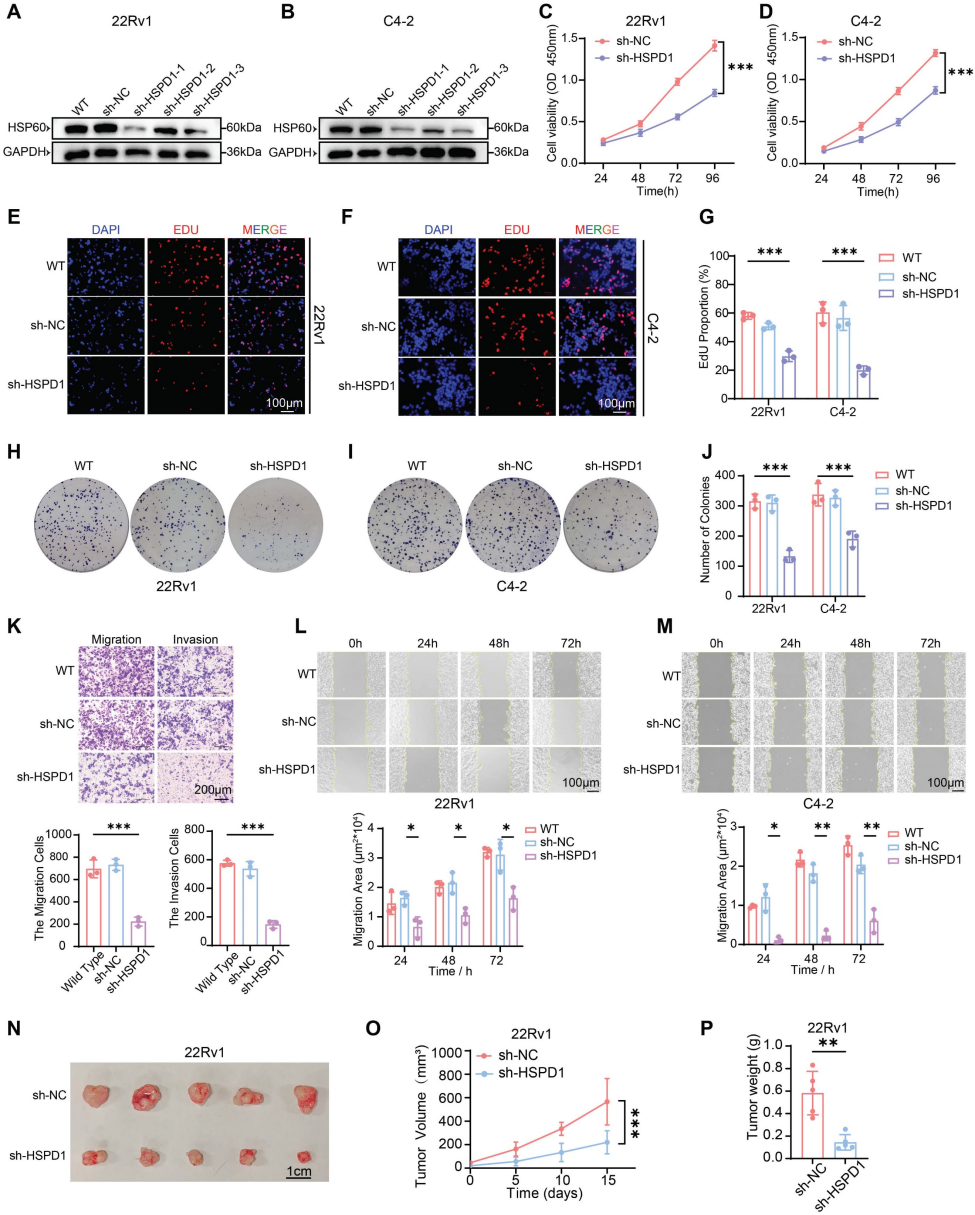
** HSP60 promotes proliferation and metastasis of PCa cells. (A)** Western blot analysis of HSP60 protein expression in 22Rv1 cells following lentivirus-mediated *HSPD1* silencing.** (B)** Western blot detection of HSP60 in C4-2 cells post-*HSPD1* knockdown.** (C)** CCK-8 assay measuring viability of 22Rv1 cells at 24 h, 48 h, 72 h, and 96 h. **(D)** CCK-8 assay measuring viability of C4-2 cells at 24 h, 48 h, 72 h, and 96 h. **(E)** EdU proliferation assay in 22Rv1 cells. **(F)** EdU proliferation assay in C4-2 cells. **(G)** Quantification of EdU-positive cells across experimental groups. **(H)** Colony formation assay of 22Rv1 cells.** (I)** Colony formation assay of C4-2 cells.** (J)** Quantitative comparison of colony numbers among groups.** (K)** Transwell migration/invasion assays with semiquantitative analysis (top: representative images; bottom: statistical plots). **(L)** Wound healing assay (top) and migration area quantification (bottom) in 22Rv1 cells.** (M)** Wound healing assay (top) and migration area quantification (bottom) in C4-2 cells. **(N)** Excised subcutaneous 22Rv1 xenograft tumors from nude mice (sh-NC vs. sh-*HSPD1*, n = 5). **(O)** Longitudinal monitoring of tumor volume in 22Rv1 xenograft models.** (P)** Terminal tumor weight comparison between control and experimental groups. Statistical analysis is performed using two-sided t-test **(G), (J), (K), (L), (M), (P)** and tow-way ANOVA in **(C), (D), (O);** Means ± SD, *P < 0.05; **P < 0.01; ***P < 0.001.

To mechanistically delineate HSP60's role in PCa progression, we employed lentiviral-mediated genetic silencing and overexpression of *HSPD1* to systematically interrogate its impact on cellular functional dynamics. We first established stable 22Rv1 and C4-2 cell lines with *HSPD1* knockdown or overexpression **(Fig. 2A-B, Fig. S3A-C)**. Given the most robust knockdown efficiency of sh-*HSPD1*-3 (about 80% reduction vs. sh-NC), this construct was selected for subsequent functional experiments. To systematically evaluate *HSPD1*'s functional impact, we deployed series assays: CCK-8 viability, EdU proliferation, and colony formation for proliferative profiling; wound healing and Transwell migration/invasion for metastatic potential. Bidirectional perturbations (knockdown/overexpression) were validated in 22Rv1 and C4-2 models. Genetic knockdown of *HSPD1* robustly suppressed PCa cell viability **(Fig. 2C-D)**, EdU+ proliferative fractions **(Fig. 2E-G)**, and colony formation potential **(Fig. 2H-J)**, while concurrently impairing migratory/invasive capacities **(Fig. 2K)** and wound-healing kinetics **(Fig. 2L-M)**. Conversely, *HSPD1* overexpression enhanced the proliferation and metastasis of PCa cells **(Fig. S3D-H)**.

To establish *HSPD1*'s *in vivo* oncogenic role, we generated subcutaneous 22Rv1 xenografts in nude mice. In the sh-*HSPD1* knockdown model, initial tumor formation was first observed on day 15 (designated as day 0 for measurement), while tumor growth kinetics revealed robust attenuation of sh-*HSPD1* tumor progression versus sh-NC controls, culminating in a 72.3% reduction in terminal tumor volume (*P* < 0.001) **(Fig. 2O)**. Terminal tumor measurements confirmed an 80.6% decrease in sh-*HSPD1* xenograft mass (*P* < 0.01) **(Fig. 2P)**, with gross morphological analysis demonstrating markedly diminished tumor sizes in the knockdown group **(Fig. 2N)**. Conversely, in the *HSPD1* overexpression model, initial tumor formation occurred earlier, first observed on day 10 (designated as day 0), and overexpression of *HSPD1* significantly accelerated *in vivo* tumor growth** (Fig. S3I-K).** Collectively, these *in vivo* findings demonstrated that knockdown of *HSPD1* attenuated while its overexpression promoted 22Rv1 PCa cell proliferation in xenograft models.

### Integrated multi-omics analyses revealed that *HSPD1* promotes glycolysis of PCa

**Figure 3:**
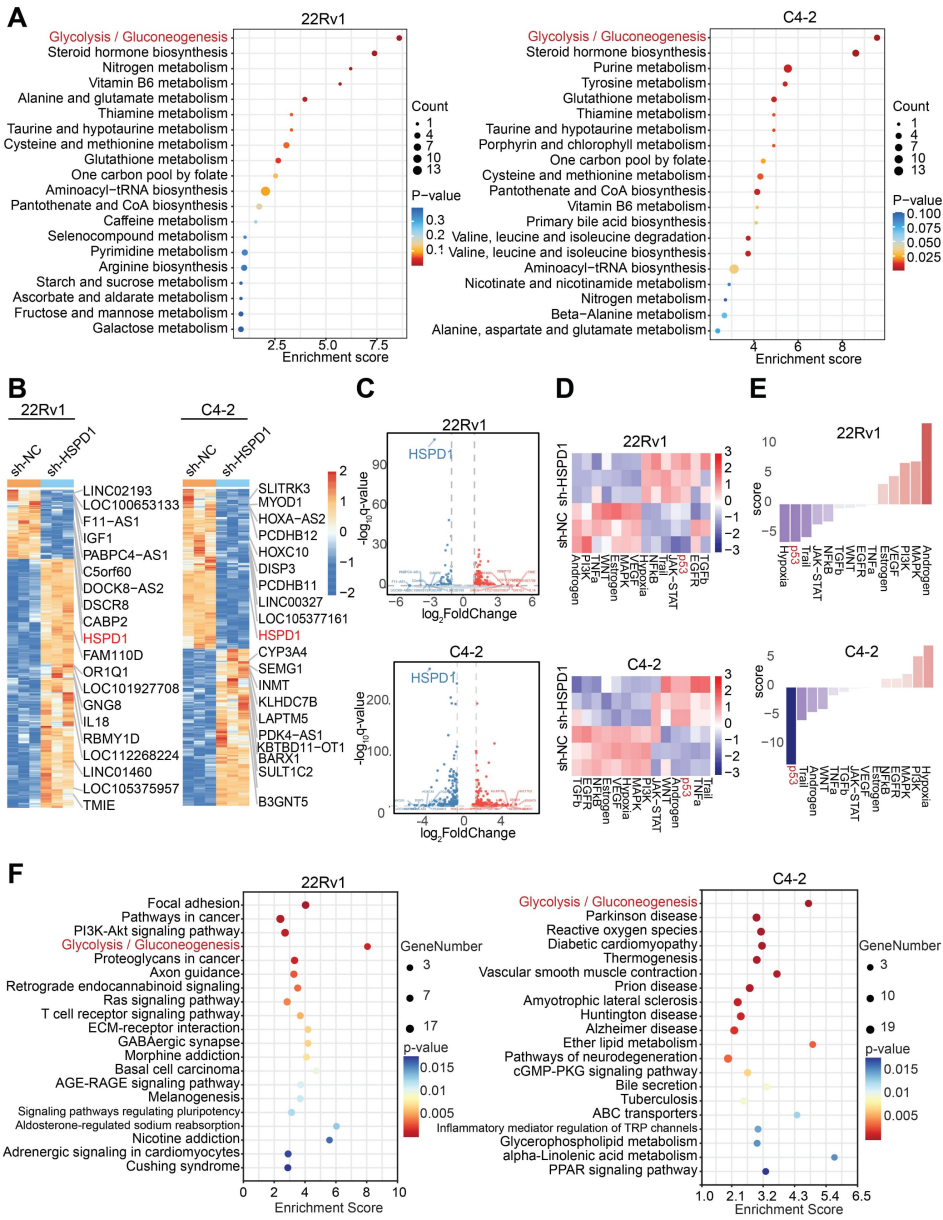
**Integrated multi-omics analyses revealed that *HSPD1* promotes glycolysis of PCa. (A)** KEGG enrichment analysis of differential metabolites in 22Rv1 and C4-2 cells. **(B)** Hierarchical clustering of differentially expressed genes in 22Rv1 and C4-2 cells. **(C)** Volcano plot visualizing differentially expressed genes in 22Rv1 and C4-2 cells. **(D)** Heatmap of pathway activity scores across samples and pathways in 22Rv1 and C4-2 cells.** (E)** Pathway activity scores in 22Rv1 and C4-2 cells. **(F)** KEGG enrichment analysis of transcriptomic differences in 22Rv1 and C4-2 cells.

Given the high complexity and systemic nature of biological processes, single-omics studies exhibit significant limitations in comprehensively elucidating developmental patterns of biological systems 28. Therefore, this study integrated metabolomics and transcriptomics data to jointly investigate the downstream mechanisms. To delineate *HSPD1*'s metabolic regulatory role, we performed untargeted metabolomics profiling on *HSPD1*-knockdown (sh-*HSPD1*) and control (sh-NC) 22Rv1/C4-2 cells. Quality control (QC) was rigorously performed to ensure data reliability. The total ion chromatogram (TIC) overlay of QC samples showed high stability and reproducibility in both positive and negative ion modes** (Fig. S4A-B)**. Principal component analysis (PCA) and Orthogonal Partial Least Squares-Discriminant Analysis (OPLS-DA) revealed pronounced intergroup metabolic divergence with minor intra-group variability **(Fig. S4C-D)**. Volcano plots identified 59 and 158 significantly altered metabolites (|log2FC| > 2, VIP > 1) in 22Rv1 and C4-2 cells, respectively **(Fig. S4E)**, while hierarchical clustering heatmaps delineated group-specific metabolic signatures **(Fig. S4F)**. Kyoto Encyclopedia of Genes and Genomes (KEGG) pathway enrichment analysis of differential metabolites pinpointed glycolysis/gluconeogenesis as the most significantly perturbed pathway **(Fig. 3A).** To explore *HSPD1*-regulated downstream pathways, we conducted transcriptomic profiling of sh-NC versus sh-*HSPD1* 22Rv1 and C4-2 cells. **Fig. S4G-H** showed the distribution of gene expression levels in the two cell lines respectively. Hierarchical clustering heatmaps identified the top 10 upregulated and downregulated genes—including *HSPD1* **(Fig. 3B)**. Volcano plots confirmed *HSPD1* as the most significantly downregulated gene in both cell lines **(Fig. 3C)**, validating knockdown efficiency. KEGG pathway analysis of differentially expressed genes revealed convergently enriched glycolysis/gluconeogenesis signaling in 22Rv1 and C4-2 cell lines **(Fig. 3F)**, mechanistically linking *HSPD1* loss to glycolytic flux suppression. Based on integrated analyses of the aforementioned metabolomics and transcriptomics results, this study confirmed that *HSPD1* plays a critical regulatory role in the glycolytic pathway.

### *HSPD1* promotes the malignant progression of PCa cells by enhancing glycolysis

**Figure 4:**
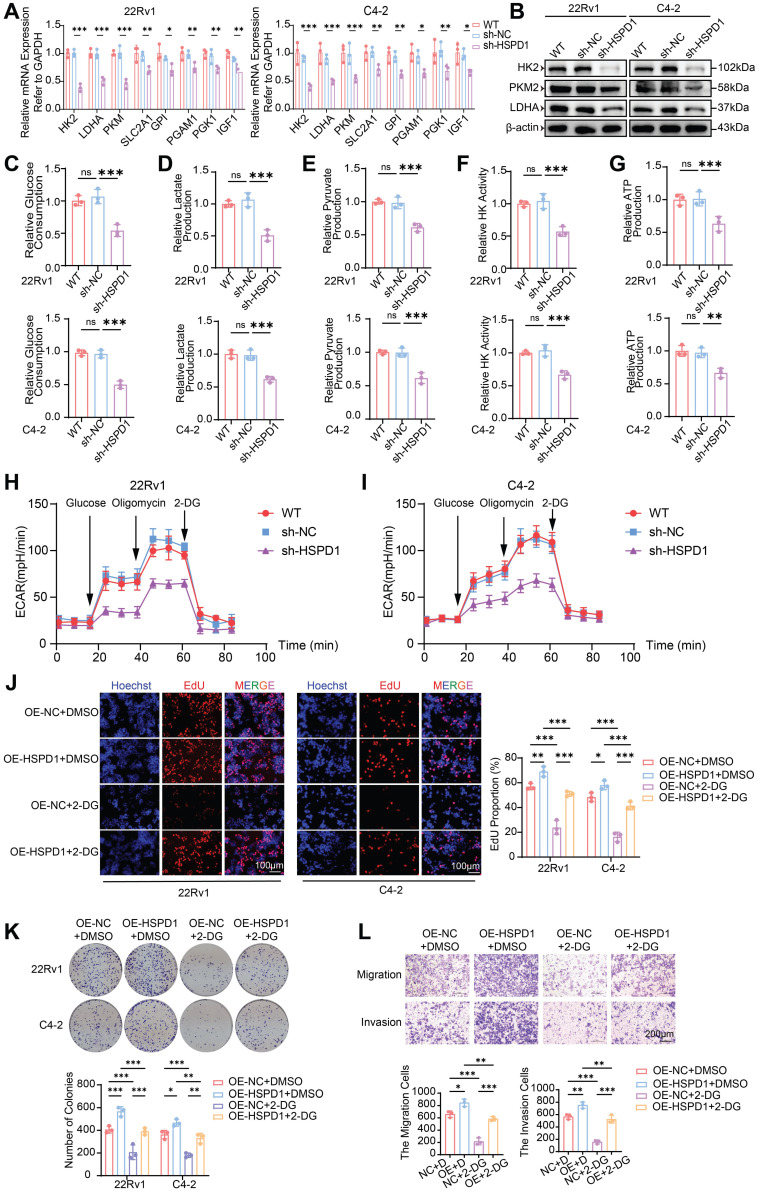
***HSPD1* promotes the malignant progression of PCa cells by enhancing glycolysis. (A)** qPCR analysis of glycolysis-related gene expression in 22Rv1 and C4-2 cells following *HSPD1* knockdown. **(B)** Western blot of glycolytic enzymes in 22Rv1 and C4-2 cells following *HSPD1* knockdown. **(C)** Measurement of relative glucose consumption in *HSPD1*-silenced 22Rv1 and C4-2 cells. **(D)** Measurement of relative lactate production in *HSPD1*-silenced 22Rv1 and C4-2 cells.** (E)** Measurement of relative pyruvate production in *HSPD1*-silenced 22Rv1 and C4-2 cells. **(F)** Measurement of relative HK activity in *HSPD1*-silenced 22Rv1 and C4-2 cells. **(G)** Measurement of relative ATP production in *HSPD1*-silenced 22Rv1 and C4-2 cells. **(H)** Measurement of ECAR in *HSPD1*-silenced 22Rv1 cells. **(I)** Measurement of ECAR in *HSPD1*-silenced C4-2 cells.** (J)** EdU proliferation assay (left: representative staining; right: quantitative analysis) in 22Rv1 and C4-2 cells across experimental conditions. **(K)** Colony formation assays (top) and quantitative comparison (bottom) in 22Rv1 versus C4-2 cells under indicated treatments. **(L)** Transwell migration/invasion assays with semiquantitative analysis (top: representative images; bottom: statistical plots) under indicated treatments. Statistical analysis is performed using two-sided t-test **(A), (C), (D), (E), (F), (G), (J), (K), (L);** Means ± SD, *P < 0.05; **P < 0.01; ***P < 0.001.

To explore *HSPD1*'s regulatory hierarchy within the glycolytic network, co-expression analysis across four independent cohorts revealed robust positive correlations between *HSPD1* and core glycolytic genes (*HK2*, *LDHA*, *PKM*, *SLC2A1*, *GPI*, *PGAM1*, *PGK1*) **(Fig. S4I)**. Targeted qPCR quantification in *HSPD1*-knockdown 22Rv1/C4-2 cells demonstrated significant downregulation of these glycolytic genes versus WT and sh-NC controls (*P* < 0.05), with *HK2*, *LDHA*, and *PKM* exhibiting the most pronounced suppression **(Fig. 4A)**. Western blot validation confirmed obvious reductions in HK2, PKM2, and LDHA abundance following both pharmacological HSP60 inhibition (Nonactin) and genetic *HSPD1* knockdown **(Fig. S5A, Fig. 4B)**, whereas *HSPD1* overexpression conversely amplified their expression **(Fig. S5B)**.

To comprehensively interrogate *HSPD1*'s functional impact on glycolytic flux, we quantified multiple metabolic nodes: glucose consumption, lactate/pyruvate accumulation, HK activity, and ATP production. *HSPD1* knockdown in 22Rv1 and C4-2 cells induced profound glycolytic suppression, marked by reduced glucose uptake (54% and 50%, *P* < 0.001) **(Fig. 4C)**, diminished lactate (51% and 62%,* P* < 0.001) **(Fig. 4D)** and pyruvate (61% and 62%, *P* < 0.001) **(Fig. 4E)** output, attenuated HK activity (57% and 67%, *P* < 0.001) **(Fig. 4F)**, and depleted ATP production (63% and 67%, *P* < 0.01) **(Fig. 4G)**. Conversely, *HSPD1* overexpression amplified these parameters by 1.3-2.2-fold **(Fig. S5C-G)**. Moreover, the seahorse analysis indicated that *HSPD1* knockdown significantly reduces glycolytic capacity **(Fig. 4H-I)**, while its overexpression produces the opposite effect **(Fig. S5H-I)**. This multi-layered interrogation—spanning substrate utilization (glucose), pathway intermediates (lactate/pyruvate), rate-limiting enzymatic activity (HK), and bioenergetic output (ATP)—definitively establishes *HSPD1* as a central orchestrator of glycolytic reprogramming in PCa.

To determine whether *HSPD1* drives malignant phenotypes through glycolytic reprogramming, we treated *HSPD1*-overexpressing (OE-*HSPD1*) PCa cells with the glycolysis inhibitor 2-DG. *HSPD1* overexpression enhanced cell viability (CCK-8), proliferation (EdU), clonogenicity, migration and invasion (Transwell), while 2-DG treatment suppressed these phenotypes. Critically, *HSPD1* overexpression partially rescued 2-DG-induced suppression across all assays **(Fig. S5J-K, Fig. 4J-L)**. Collectively, these functional rescue experiments demonstrate *HSPD1* sustains PCa proliferation and metastasis by glycolytic dependency, as its overexpression counteracts 2-DG-induced metabolic suppression. This mechanistic linkage positions *HSPD1* as a critical part of the Warburg effect in prostate oncogenesis.

### HSP60 promotes glycolysis of PCa cells by suppressing p53 activity

To investigate downstream pathway alterations following *HSPD1* suppression, we performed functional enrichment analysis of transcriptomic data using the decoupleR algorithm, combined with PROGENy pathway activity profiling to systematically evaluate key signaling cascades. Heatmap visualization revealed lineage-specific pathway activation: p53, Trail, NF-κB, and hypoxia pathways dominated in 22Rv1 cells, while p53, Trail, TNF-α, and androgen signaling were prioritized in C4-2 cells **(Fig. 3D)**. t-score-weighted pathway analysis further identified p53 and Trail as the most robustly activated pathways across both models **(Fig. 3E)**. Notably, *HSPD1* silencing induced profound p53 pathway activation, a master regulator of apoptosis and genomic stability. These coordinated findings suggest *HSPD1* loss triggers tumor-suppressive stress responses by activating p53 and promoting apoptosis, potentially counterbalancing its oncogenic glycolytic functions.

**Figure 5:**
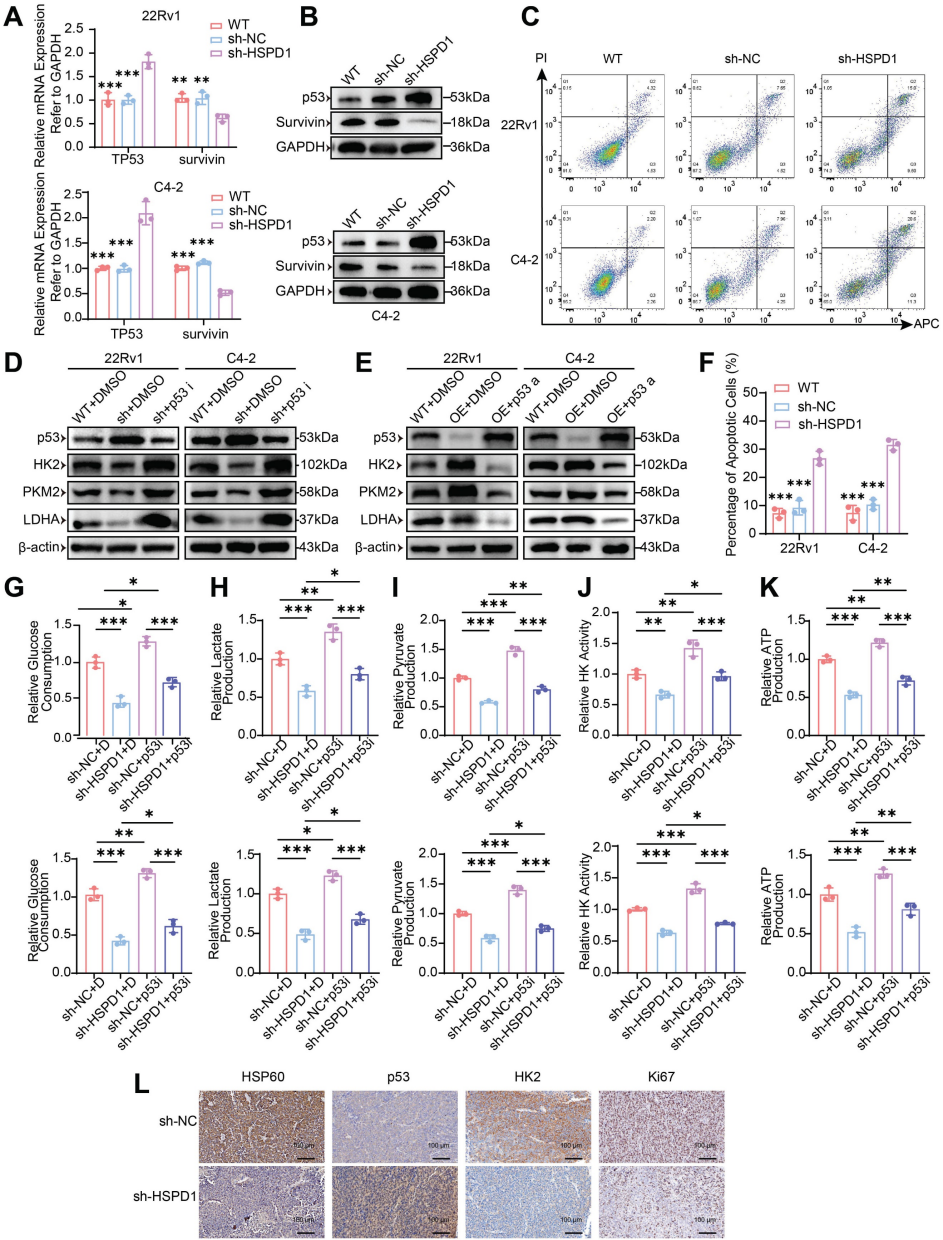
** HSP60 promotes glycolysis of PCa cells by suppressing p53 activity. (A)** qPCR quantification of TP53 and survivin (BIRC5) mRNA levels in *HSPD1*-knockdown 22Rv1 and C4-2 cells. **(B)** Western blot analysis of p53 and Survivin protein expression in *HSPD1*-knockdown 22Rv1 and C4-2 cells. **(C)** Flow cytometry apoptosis assay in *HSPD1*-knockdown 22Rv1 and C4-2 cells. **(D)** Western blot detection of p53 and glycolytic enzymes in wild-type and *HSPD1*-knockdown cells treated with p53 inhibitor. **(E)** Western blot detection of p53 and glycolytic enzymes in wild-type and *HSPD1*-overexpressing cells treated with p53 agonist. **(F)** Quantitative comparison of apoptotic rates among different groups. **(G)** p53 inhibitor-mediated change of glucose consumption in *HSPD1*-knockdown 22Rv1 and C4-2 cells. **(H)** p53 inhibitor-mediated change of lactate production in *HSPD1*-knockdown 22Rv1 and C4-2 cells. **(I)** p53 inhibitor-mediated change of pyruvate production in *HSPD1*-knockdown 22Rv1 and C4-2 cells. **(J)** p53 inhibitor-mediated change of HK activity in *HSPD1*-knockdown 22Rv1 and C4-2 cells. **(K)** p53 inhibitor-mediated change of ATP production in *HSPD1*-knockdown 22Rv1 and C4-2 cells. **(L)** IHC staining of HSP60, p53, HK2, and Ki67 in xenograft tumor sections. Statistical analysis is performed using two-sided t-test **(A), (F), (G), (H), (I), (J), (K);** Means ± SD, *P < 0.05; **P < 0.01; ***P < 0.001.

Studies have shown that HSP60 forms a chaperone complex with p53 that suppresses its activity in cytosolic and mitochondrial compartments, thereby modulating apoptotic signaling 29, 30. Survivin, an anti-apoptotic effector within the inhibitor of apoptosis proteins (IAPs) family, counteracts programmed cell death 31. Bidirectional perturbations of *HSPD1* (knockdown and overexpression) combined with pharmacological HSP60 inhibition (Nonactin) revealed an inverse regulatory relationship between *HSPD1* and the p53-survivin axis, which is consistent with research reports 32. *HSPD1* silencing or HSP60 inhibition elevated *TP53* mRNA and p53 protein while suppressing survivin expression **(Fig. 5A-B, Fig. S6B, D)**. Conversely, *HSPD1* overexpression reduced p53 levels and amplified survivin **(Fig. S6A, C)**. To assess HSP60's role in apoptotic regulation, we quantified apoptosis via Annexin V-APC/PI dual staining and flow cytometry. Both pharmacological HSP60 inhibition and *HSPD1* knockdown significantly increased apoptotic fractions in 22Rv1 and C4-2 cells **(Fig. 5C, F, Fig. S6E, G)**. Conversely, *HSPD1* overexpression reduced apoptosis compared to controls **(Fig. S6F, H)**. Mechanistically, *HSPD1* sustains tumor cell survival by suppressing pro-apoptotic signaling through the p53-survivin axis, positioning HSP60 as a therapeutically actionable node to overcome apoptotic resistance.

To determine whether HSP60 regulates glycolytic enzymes via p53, we modulated p53 activity in 22Rv1/C4-2 cells using Pifithrin-α hydrobromide (inhibitor) and Nutlin-3a (activator). *HSPD1* knockdown (sh-*HSPD1* + DMSO) significantly elevated p53 protein while suppressing HK2, PKM2, and LDHA compared to control (WT+DMSO). p53 inhibition (sh-*HSPD1* + p53i) reversed these effects, restoring glycolytic enzyme expression **(Fig. 5D)**. Conversely, *HSPD1* overexpression reduced p53 and upregulated glycolytic enzymes, which were suppressed by p53 activation **(Fig. 5E)**. These bidirectional experiments establish HSP60 as a metabolic switch that drives glycolytic reprogramming by antagonizing p53-mediated repression of HK2, PKM2, and LDHA.

To dissect HSP60's p53-mediated glycolytic regulation, we performed phenotypic assays in *HSPD1*-perturbed 22Rv1 and C4-2 cells under p53 modulation. *HSPD1* knockdown reduced glucose consumption (vs. sh-NC+DMSO, *P* < 0.001), which was partially rescued by p53 inhibition (sh-*HSPD1*+p53i; *P* < 0.05) **(Fig. 5G)**. Conversely, *HSPD1* overexpression increased glucose uptake (vs. OE-NC+DMSO, *P* < 0.05), attenuated by p53 activation (OE-*HSPD1*+p53a; *P* < 0.001) **(Fig. S6I)**. Parallel trends were observed for lactate/pyruvate production, HK activity, and ATP production. *HSPD1* knockdown suppressed glycolytic flux (*P* < 0.01), partially reversed by p53 inhibition (*P* < 0.05) **(Fig. 5H-K)**. *HSPD1* overexpression enhanced glycolysis (*P* < 0.05), counteracted by p53 activation (*P* < 0.05) **(Fig. S6J-M)**. These multi-layered analyses mechanistically resolve HSP60's role in promoting glycolysis via p53 inactivation, enabling metabolic reprogramming in PCa.

In addition, IHC analysis of tumor xenografts revealed coordinated dysregulation of key biomarkers in *HSPD1*-knockdown tumors: HSP60, HK2, and Ki67 expression were significantly reduced, while p53 levels increased **(Fig. 5L)**. These *in vivo* findings align with prior *in vitro* data, validating HSP60's role in sustaining PCa proliferation through p53-mediated glycolytic activation.

### Construction of siRNA@EVs and verification of their basic characteristics

**Figure 6:**
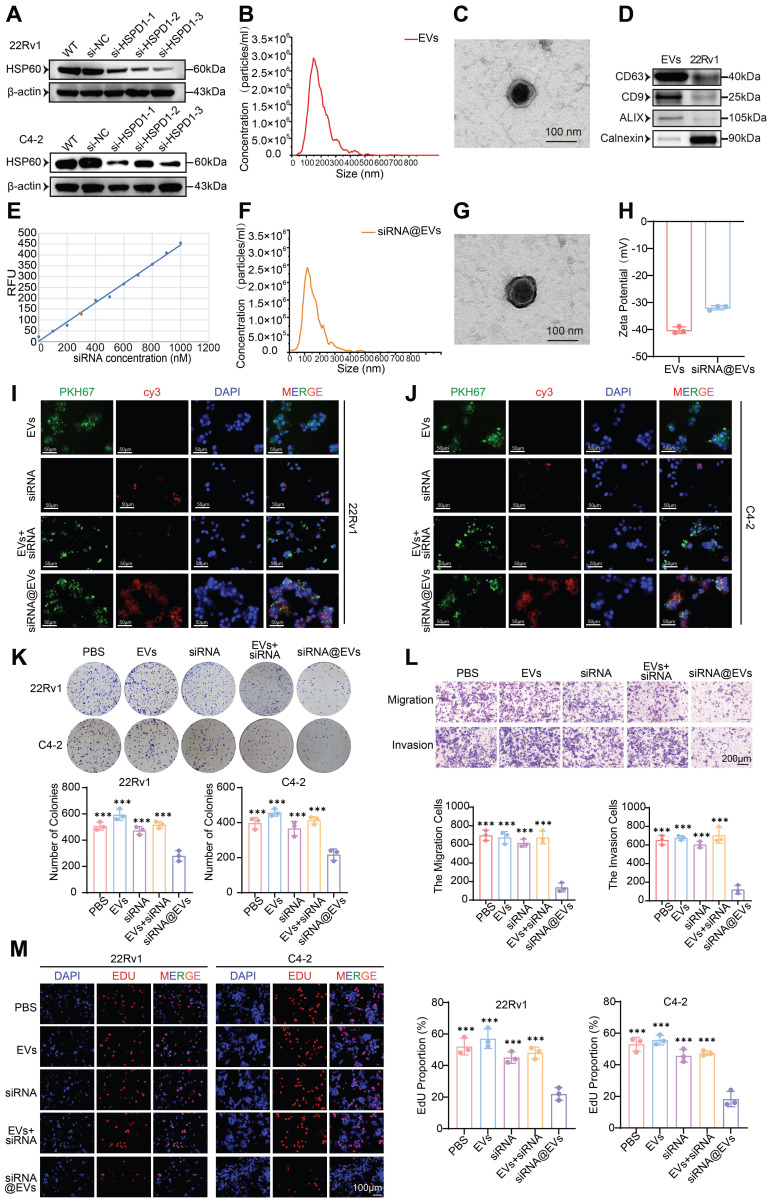
** Construction of siRNA@EVs, verification of their basic characteristics and effect. (A)** Western blot validation of HSP60 expression in 22Rv1 and C4-2 cells following si-*HSPD1* transfection. **(B)** Nanoparticle tracking analysis (NTA) of 22Rv1-derived EVs.** (C)** TEM characterization of EVs morphology. **(D)** Western blot detection of EVs biomarkers.** (E)** Standard curve correlating siRNA concentrations with fluorescence intensity. **(F)** NTA of electroporated siRNA@EVs. **(G)** TEM visualization of siRNA@EVs.** (H)** Zeta potential analysis of EVs and siRNA@EVs. **(I)** Immunofluorescence assessment of siRNA@EVs uptake efficiency in 22Rv1 cells. **(J)** Immunofluorescence assessment of siRNA@EVs uptake efficiency in C4-2 cells.** (K)** Colony formation assays (top) and quantitative comparison (bottom) in 22Rv1 and C4-2 cells under therapeutic interventions. **(L)** Transwell migration/invasion assays with semiquantitative analysis (top: representative images; bottom: statistical plots) under therapeutic interventions. **(M)** EdU proliferation assay (left: representative staining; right: quantitative analysis) in 22Rv1 and C4-2 cells under therapeutic interventions. Statistical analysis is performed using two-sided t-test **(K), (L), (M);** Means ± SD, *P < 0.05; **P < 0.01; ***P < 0.001.

Firstly, the transfection efficiency of si-*HSPD1* was evaluated. si-*HSPD1*-3 demonstrated optimal *HSPD1* mRNA silencing **(Fig. S7A)** and HSP60 protein suppression **(Fig. 6A)**, validating its use for downstream assays.

The biophysical and molecular properties of EVs isolated from 22Rv1 cells were systematically validated: NTA revealed an average size of 148 nm **(Fig. 6B)**, while TEM confirmed characteristic spherical morphology with intact bilayers **(Fig. 6C)**. Western blotting verified robust expression of exosomal markers CD63, CD9, and ALIX **(Fig. 6D)**. Post-electroporation siRNA loading achieved 42% encapsulation efficiency **(Fig. 6E)** without compromising EV integrity, as evidenced by preserved particle size (134 nm; NTA,** Fig. 6F**), unaltered ultrastructure (TEM, **Fig. 6G**), and consistent surface charge (Zeta potential,** Fig. 6H**). This characterization confirms the suitability of siRNA@EVs for functional interrogation while maintaining native vesicle architecture. Immunofluorescence imaging demonstrated efficient cytoplasmic internalization of PKH67-labeled EVs (green) and Cy3-tagged siRNA (red) in 22Rv1 and C4-2 cells, with signal localization distinct from DAPI-stained nuclei. While free siRNA or siRNA+EVs mixtures showed minimal cytoplasmic uptake, siRNA@EVs exhibited robust delivery efficiency **(Fig. 6I-J)**. These results confirm EVs as superior nanocarriers for PCa-targeted siRNA delivery, enabling precise cytoplasmic payload release.

### siRNA@EVs suppress malignant phenotypes in PCa

siRNA@EVs profoundly inhibited PCa proliferation across functional assays: CCK-8 viability assays demonstrated 13-31% reduction in PCa cell activity (*P* < 0.05) **(Fig. S7B)**, while colony formation capacity was similarly attenuated in siRNA@EVs-treated cells (*P* < 0.001) **(Fig. 6K)**. EdU assays revealed 58-65% suppression of DNA replication (*P* < 0.001) **(Fig. 6M)**. Transwell migration/invasion assays demonstrated siRNA@EVs' anti-metastatic efficacy, reducing migration/invasion cell counts by 80-82% compared to PBS, EVs, free siRNA, or siRNA+EVs mixtures (*P* < 0.001) **(Fig. 6L)**. Notably, comparative analysis with existing therapeutics revealed that siRNA@EVs exhibited superior cancer cell-specific killing compared to enzalutamide, while unlike docetaxel, it showed no significant cytotoxicity toward normal prostate epithelial cells** (Fig. S7C)**. These findings establish siRNA@EVs as a potential safe therapeutic approach, simultaneously targeting proliferative and metastatic vulnerabilities in PCa.

### siRNA@EVs curtail PCa proliferation via p53-mediated glycolytic reprogramming

**Figure 7:**
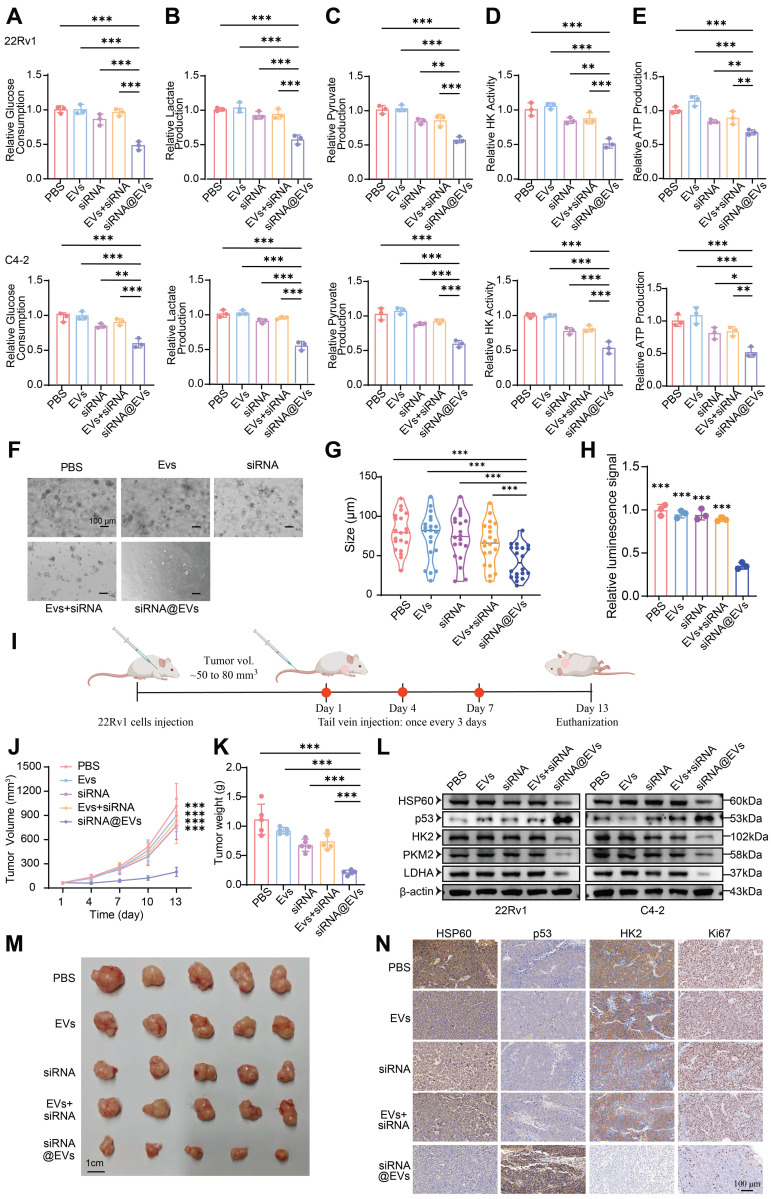
** siRNA@EVs curtail PCa proliferation via p53-mediated glycolytic reprogramming. (A)** Relative glucose consumption of 22Rv1 and C4-2 cells under different therapeutic interventions. **(B)** Relative lactate production of 22Rv1 and C4-2 cells under different therapeutic interventions.** (C)** Relative pyruvate production of 22Rv1 and C4-2 cells under different therapeutic interventions. **(D)** Relative HK activity of 22Rv1 and C4-2 cells under different therapeutic interventions. **(E)** Relative ATP production of 22Rv1 and C4-2 cells under different therapeutic interventions. **(F)** Representative patient-derived organoid images following 14-day exposure to indicated treatment. Scale bar, 100 μm.** (G)** Quantification analysis of organoid size (n =20).** (H)** Relative ATP levels (Luminescence signal) of organoid treated with indicated treatment were shown. **(I)** Schematic of subcutaneous xenograft establishment and therapeutic regimen. **(J)** Longitudinal monitoring of 22Rv1-derived tumor volume progression. **(K)** Terminal tumor weight comparison among treatment groups. **(L)** Macroscopic presentation of excised 22Rv1 xenograft tumors. **(M)** Western blot of HSP60, p53, and glycolytic enzyme expression of 22Rv1 and C4-2 cells under therapeutic conditions.** (N)** IHC evaluation of HSP60/p53/HK2/Ki67 biomarkers in xenograft tumor sections. Statistical analysis is performed using two-sided t-test **(A), (B), (C), (D), (E), (G), (H), (K)** and tow-way ANOVA in **(J);** Means ± SD, *P < 0.05; **P < 0.01; ***P < 0.001.

Systematic evaluation of siRNA@EVs' metabolic impact revealed robust suppression of glycolytic flux in PCa cells. siRNA@EVs treatment reduced glucose consumption by 39-52% in 22Rv1 and C4-2 cells versus PBS, EVs, free siRNA, or siRNA+EVs controls (*P* < 0.01) **(Fig. 7A)**. Parallel reductions were observed in lactate production (*P* < 0.001) **(Fig. 7B)**, pyruvate production (*P* < 0.01) **(Fig. 7C)**, HK activity (*P* < 0.01) **(Fig. 7D)**, and ATP production (*P* < 0.05) **(Fig. 7E)**. This concerted attenuation of key metabolic nodes demonstrates siRNA@EVs effectively disrupts glycolytic reprogramming.

To substantiate these results, we conducted functional validation using patient-derived organoid (PDO) models. Consistent with the cellular phenotype observed *in vitro*, siRNA@EVs administration markedly suppressed organoid proliferation, demonstrating superior efficacy compared to individual therapeutic agents** (Fig. 7F-H)**. These data underscore the translational potential of siRNA@EVs as a novel therapeutic strategy for prostate cancer intervention.

To evaluate siRNA@EVs' anti-tumor efficacy *in vivo*, subcutaneous 22Rv1 xenografts were established in nude mice. Upon reaching 50-80 mm³, nude mice received tail vein injections of PBS, EVs, free siRNA, siRNA+EVs, or siRNA@EVs **(Fig. 7I)**. Longitudinal monitoring revealed siRNA@EVs significantly suppressed tumor growth kinetics (*P* < 0.001) **(Fig. 7J)**, culminating in reduced terminal tumor mass **(Fig. 7K)** and volume **(Fig. 7M)**. No significant intergroup differences were observed among control cohorts. H&E staining confirmed gross histopathological integrity across major organs **(Fig. S7D)**, supported by normal serum biochemical parameters** (Fig. S7E)** and absence of behavioral abnormalities. These findings demonstrate siRNA@EVs' therapeutic advantage: potent tumor suppression coupled with favorable safety.

Mechanistic studies revealed siRNA@EVs suppress PCa proliferation through coordinated modulation of the HSP60/p53-glycolysis axis. In 22Rv1 and C4-2 cells, siRNA@EVs treatment significantly reduced HSP60 protein levels while elevating p53 expression, concomitant with significant downregulation of glycolytic enzymes HK2, PKM2, and LDHA **(Fig. 7L)**. IHC analysis of xenograft tissues confirmed concordant molecular changes: siRNA@EVs-treated tumors exhibited significantly reduced HSP60 and HK2 expression with concomitant p53 activation and decreased Ki67+ proliferation **(Fig. 7N)**. Therapeutic efficacy arises through some interlinked mechanisms: *HSPD1* silencing reduces HSP60 protein levels, thereby derepressing p53-mediated tumor suppression, p53 activation transcriptionally represses glycolytic effectors HK2, PKM2, and LDHA to impair Warburg metabolism, and metabolic control of proliferation, establishing siRNA@EVs as precision nanotherapy targeting PCa's metabolic-transcriptional axis.

## Discussion

PCa remains a leading cause of cancer-related morbidity and mortality in men 33, necessitating the identification of novel therapeutic targets and precision strategies 34. This study systematically elucidates the oncogenic role of HSP60 in PCa progression, delineates its mechanism in metabolic reprogramming via p53 suppression, and pioneers an EVs-based siRNA delivery system (siRNA@EVs) as a promising therapeutic approach. Our findings establish HSP60 as a pivotal orchestrator of PCa progression, metastasis, and metabolic reprogramming, while delineating an actionable strategy to disrupt its oncogenic dependencies through precision RNA interference.

### HSP60 as a clinically relevant therapeutic target

Integrative analyses of DepMap, TCGA, and HPA databases established *HSPD1* (encoding HSP60) as a PCa (PCa)-specific therapeutic target through a multidimensional strategy that overcomes the limitations of single-platform approaches. While DepMap's CRISPR-based functional genomics identified *HSPD1* as a lineage-specific dependency gene, TCGA clinical cohorts revealed its overexpression in tumors and association with advanced GS, T-stage, and biochemical recurrence. HPA further validated cancer-specific HSP60 protein enrichment and mitochondrial localization, distinguishing it from benign tissues. This "cell-to-clinic" integration resolved critical gaps in target prioritization: DepMap's short-term viability screens, which prioritize acute survival genes 35 (e.g., cell cycle regulators), failed to capture *HSPD1*'s context-dependent vulnerabilities. Instead, *HSPD1* ablation triggered delayed cytotoxicity via mitochondrial proteostasis collapse and metabolic dysregulation—phenotypes amplified under androgen deprivation, a hallmark of advanced PCa^36^. These findings align with HSP60's emerging roles in stress adaptation and microenvironment remodeling, mechanisms undetectable in conventional assays but critical for tumor resilience.

Functional validation across *in vitro* and *in vivo* models confirmed HSP60's direct pro-tumorigenic role. Pharmacological inhibition (Nonactin) and genetic silencing of *HSPD1* suppressed PCa proliferation, migration, and xenograft growth, while overexpression exacerbated malignant phenotypes. This bidirectional consistency underscores HSP60's centrality in PCa progression, independent of off-target effects. HSP60 supports mitochondrial stability and metabolic adaptability 37, allowing tumors to cope with microenvironmental challenges like oxidative stress and nutrient scarcity 38. Multi-bioinformation data analyses and clinical evidence highlight HSP60's dual significance as both a prognostic biomarker and a potential therapeutic target.

### HSP60 drives glycolytic reprogramming via p53 inactivation

Metabolic rewiring, particularly the dominance of aerobic glycolysis (Warburg effect), is a pivotal hallmark of PCa progression 39, 40. Our integrated multi-omics approach—spanning untargeted metabolomics and transcriptomics—revealed that HSP60 (*HSPD1*) orchestrates glycolytic activation by suppressing p53-mediated metabolic regulation. Metabolomic profiling demonstrated that *HSPD1* knockdown triggered broad metabolic perturbations, including alterations in amino acids, fatty acids, and organic acid derivatives, with KEGG pathway enrichment highlighting glycolysis as the most significantly dysregulated pathway. Transcriptomic analysis further corroborated these findings, showing downregulation of glycolytic enzymes (HK2, LDHA, PKM) and enrichment of glycolysis-related gene sets. Crucially, functional validation through targeted assays confirmed that *HSPD1* silencing reduced glucose consumption, lactate/pyruvate accumulation, and ATP production—phenotypes rescued by p53 inhibition. Strikingly, *HSPD1* ablation also triggered compensatory activation of the PI3K-AKT pathway, which synergizes with residual glycolytic flux to maintain energy homeostasis 41. This dual-axis rewiring—glycolysis for rapid ATP generation and PI3K-AKT signaling for glucose uptake and survival 42—reflects a metabolic fail-safe mechanism under mitochondrial stress induced by HSP60 loss.

Mechanistically, HSP60 binds and sequesters p53 in the cytosol, preventing its nuclear translocation and transcriptional repression of glycolytic genes 43. Pharmacological inhibition or genetic silencing of *HSPD1* elevated p53 protein levels, which directly suppressed HK2, LDHA, and PKM expression, redirecting metabolism toward oxidative phosphorylation. Conversely, *HSPD1* overexpression amplified glycolysis by stabilizing cytosolic p53, thereby relieving p53-mediated transcriptional suppression of glycolytic enzymes 44. This feedforward loop—where glycolytic flux sustains HSP60 expression, and HSP60 perpetuates p53 inactivation—anchors Warburg metabolism as a self-reinforcing oncogenic driver. Notably, p53's role extends beyond transcriptional regulation; its interaction with HSP60 disrupts mitochondrial proteostasis, as evidenced by aberrant activation of steroidogenesis pathways upon *HSPD1* knockdown 45. Cholesterol side-chain cleavage enzymes (CYP11A1, HSD3B) and StAR—key mitochondrial proteins in steroid synthesis—require HSP60 for proper folding. Their dysfunction under *HSPD1* deficiency led to precursor accumulation and compensatory steroidogenic pathway activation, further linking HSP60 to metabolic plasticity.

The interplay between HSP60 and p53 also intersects with apoptotic regulation 46, 47. DecoupleR-based pathway analysis revealed coordinated activation of p53 and TRAIL signaling post-*HSPD1* knockdown, which not only suppresses glycolysis but also induces apoptosis 48. This dual pressure—metabolic collapse and pro-apoptotic signaling—explains the profound tumor-suppressive effects of HSP60 inhibition. Importantly, PI3K-AKT activation in this context may serve as a resistance mechanism, as mTORC1 stabilizes HIF-1α to sustain glycolytic enzyme expression 49, creating a metabolic "buffer" against HSP60 loss. Such crosstalk underscores the complexity of targeting HSP60 and highlights the need for combinatorial strategies to disrupt compensatory networks 50. These findings not only advance our understanding of PCa's metabolic vulnerabilities but also provide a mechanistic foundation for targeting the HSP60-p53 axis to cripple Warburg-driven malignancy.

### Therapeutic potential of siRNA@EVs

The selection of EVs as the delivery vehicle for *HSPD1*-targeting siRNA was based on their unique biological advantages, which are essential for overcoming long-standing challenges in RNAi-based therapeutics 51. First, EVs' endogenous origin confers exceptional biocompatibility and minimized immunogenicity, substantially reducing systemic toxicity risks 52. Second, their inherent tumor-homing capacity—mediated through surface molecules and physicochemical properties—enables preferential accumulation at tumor sites via both enhanced permeability and retention effects and ligand-receptor recognition 53. Most critically, EVs possess natural endosomal escape machinery that bypasses lysosomal degradation, ensuring functional cytoplasmic delivery of siRNA that overcomes the primary limitation of synthetic nanocarriers 54. This strategic combination of biosafety, targeting precision, and intracellular delivery efficiency establishes siRNA@EVs as a transformative platform for RNAi-based therapy.

In this study, *HSPD1* silencing by siRNA@EVs not only suppressed PCa proliferation and metastasis but uniquely reprogrammed tumor metabolism by dismantling the HSP60/p53/glycolysis axis. Mechanistically, HSP60 depletion liberated p53, which then transcriptionally repressed HK2, LDHA, and PKM2—core glycolytic enzymes driving the Warburg effect 55. This dual targeting of oncogenic signaling and metabolic addiction highlights a paradigm shift from single-pathway inhibition to multi-modal intervention, significantly enhancing therapeutic efficacy.

Our study pioneers a "silencing-carrier synergy" design, leveraging EVs as protective and precision-guided vehicles for siRNA, while unveiling HSP60/p53 crosstalk as a metabolic vulnerability that bridges proteostasis and glycolytic addiction in PCa. Furthermore, siRNA@EVs demonstrated clinically translatable safety with no detectable toxicity, addressing a major hurdle in RNAi therapy. By concurrently disrupting tumor survival and metabolic plasticity, this strategy transcends current RNAi limitations, establishing a blueprint for combinatorial gene-metabolism targeting in solid cancers.

### Limitations and future directions

While this study provides mechanistic and translational insights, limitations remain. First, reliance on 2D cell cultures overlooks tumor-stroma interplay; orthotopic models could better resolve HSP60's role in heterogeneous microenvironments. Second, the molecular interplay between HSP60 and p53 requires further exploration to define regulatory hierarchies. Third, despite siRNA@EVs' preclinical efficacy, scaling production necessitates optimizing EVs yield and batch consistency. Enhancing vesicle surfaces with chemical modifications could improve targeting specificity, minimize off-target effects, and reduce immune activation 56. Finally, longitudinal studies in castration-resistant models are needed to assess therapeutic durability and resistance mechanisms. These steps will bridge translational gaps while refining siRNA@EVs' clinical potential.

## Conclusion

**Figure 8:**
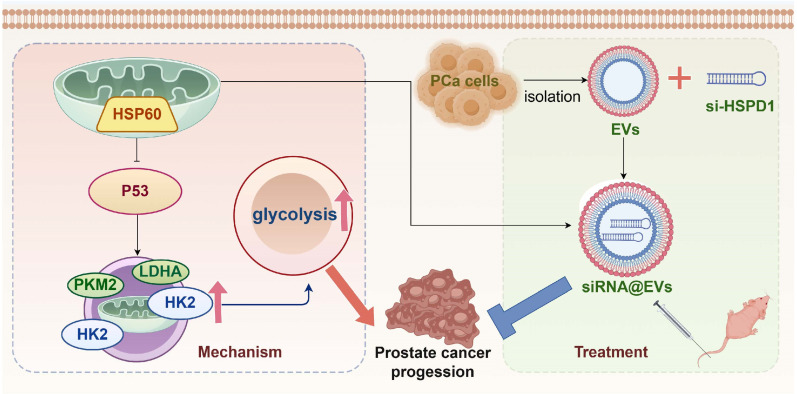
**Graphical abstract**.

HSP60 is specifically overexpressed in PCa and promotes disease progression by enhancing glycolysis via suppression of p53 activity. Conversely, siRNA@EVs inhibit PCa progression by activating p53-mediated suppression of glycolysis **(Fig. 8)**. In contrast to the limited targeted strategies currently available for PCa, this EVs-mediated siRNA delivery system represents a precision therapeutic strategy that specifically silences *HSPD1* in tumor cells, demonstrating favorable safety and efficacy profiles and offering a novel potential approach for targeted PCa therapy.

## Supplementary Material

Supplementary figures and tables.

## Abbreviations

ADT: androgen deprivation therapy
ATCC: American Type Culture Collection
BPH: benign prostatic hyperplasia
CCK-8: Cell Counting Kit-8
CI: confidence interval
CRISPR: Clustered Regularly Interspaced Short Palindromic Repeats
CRPC: castration-resistant prostate cancer
ECAR: Extracellular acidification rate
EVs: extracellular vesicles
FBS: fetal bovine serum
HK: Hexokinase
HPA: Human Protein Atlas
HSP60: heat shock protein 60
IAP: inhibitor of apoptosis protein
KEGG: Kyoto Encyclopedia of Genes and Genomes
NTA: Nanoparticle Tracking Analysis
OPLS-DA: Orthogonal Partial Least Squares-Discriminant Analysis
PCA: principal component analysis
PCa: Prostate cancer
PDO: patient-derived organoid
QC: quality control
RNA-Seq: RNA sequencing
siRNA@EVs: siRNA-loaded extracellular vesicles
TCGA: The Cancer Genome Atlas
TEM: transmission electron microscopy
TIC: total ion chromatogram
