# Supplementary material for: Targeting the HSP60/p53 Axis with Extracellular Vesicle-Delivered siRNA Reprograms Glycolysis in Prostate Cancer
Source: Int J Biol Sci. 2026 Jan 1;22(2):641–62. doi: 10.7150/ijbs.120760 (PMC12780948; doi:10.7150/ijbs.120760)
Supplement: Supplementary file 1 — Supplementary figures and tables. [file ijbsv22p0641s1.pdf]

Supplementary Figures

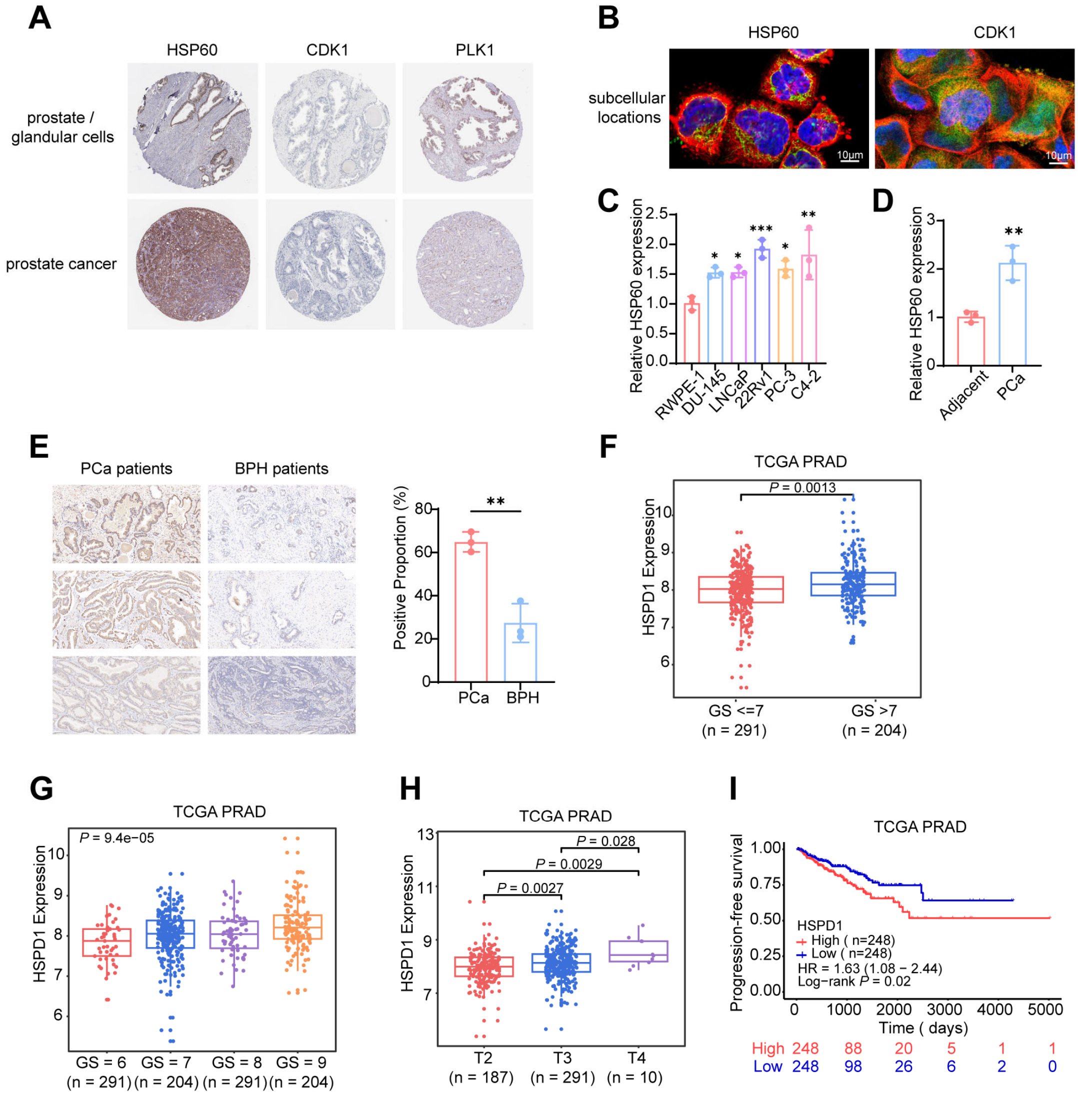

Fig. S1 HSP60 is highly expressed in PCa and is associated with PCa progression and poor prognosis

(A) Representative IHC staining of HSP60, CDK1, and PLK1 in prostate glandular versus cancer tissues (Human Protein Atlas). **(B)** Subcellular fluorescence localization in A-431 cells (HPA): nuclei (blue; Hoechst), microtubules (red; α-tubulin), and target proteins (green). **(C)** Semiquantitative analysis of HSP60 protein expression across cell lines. **(D)** Semiquantitative analysis of HSP60 protein expression across patients tissues. **(E)** Comparative IHC analysis of HSP60 expression in PCa versus BPH specimens

from Tongji Hospital. **(F)** Association between *HSPD1* expression and GS stratification (≤7 vs. >7) in TCGA-PRAD cohort. **(G)** Association between *HSPD1* expression and GS stratification (6/7/8/9) in TCGA-PRAD. **(H)** Association between *HSPD1* expression and T-stage progression (T2/T3/T4) in TCGA-PRAD. **(I)** Kaplan-Meier progression-free survival (PFS) curves stratified by *HSPD1* expression (high vs. low) in TCGA-PRAD. Statistical analysis is performed using two-sided t test **(C), (D), (E), (F), (G), (H)** and the Kruskal–Wallis test in **(I)**; Means ± SD, **P* < 0.05; ***P* < 0.01; ****P* < 0.001.

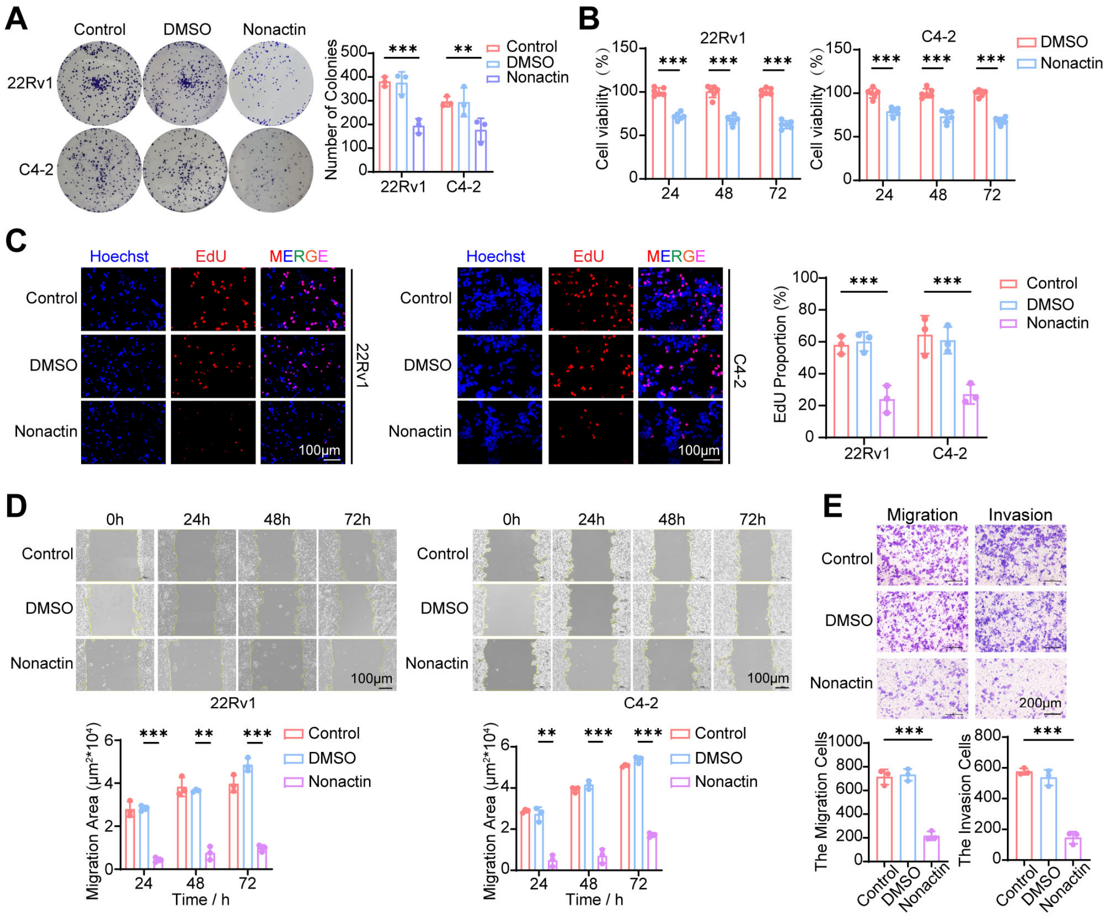

Fig. S2 HSP60 inhibitor Nonactin suppresses proliferative and metastatic capacities in PCa cells

(A) Colony formation assays (left) and quantitative comparison (right) in 22Rv1 and C4-2 cells under DMSO or Nonactin intervention. **(B)** CCK-8 assay measuring cell viability in 22Rv1 and C4-2 cells following Nonactin or DMSO treatment at 24 h, 48 h, and 72 h. **(C)** EdU proliferation assay (left: representative staining; right: quantitative analysis) in 22Rv1 and C4-2 cells under DMSO or Nonactin intervention. **(D)** Wound healing assay (top) and migration area quantification (bottom) in 22Rv1 and C4-2 cells. **(E)** Transwell migration/invasion assays with semiquantitative analysis (top: representative images; bottom: statistical plots) under DMSO or Nonactin intervention. Statistical analysis is performed using two-sided t test **(A), (B), (C), (D), (E);** Means ± SD, *$P < 0.05$; **$P < 0.01$; ***$P < 0.001$. .

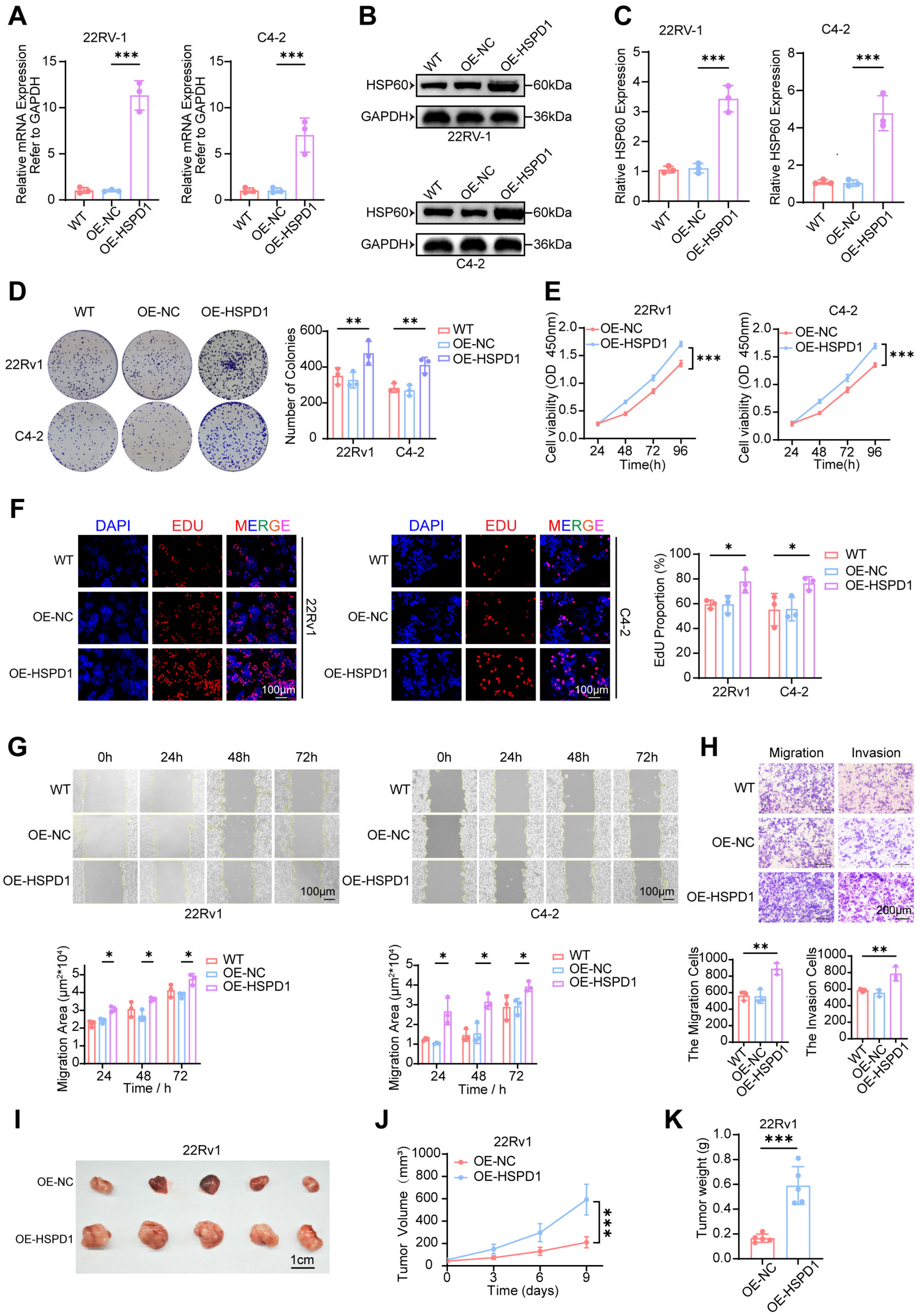

Fig. S3 *HSPD1* overexpression enhances proliferative and metastatic capacities in PCa cells

(A) qPCR analysis of *HSPD1* mRNA levels in 22Rv1 and C4-2 cells following lentivirus-mediated gene overexpression. **(B)** Western blot validation of HSP60 protein expression in *HSPD1*-overexpressing 22Rv1 and C4-2 cells. **(C)** Semiquantitative densitometric analysis of HSP60 protein levels post-*HSPD1* overexpression. **(D)** Colony formation assay (left: representative images; right: quantitative comparison) in *HSPD1*-overexpressing versus control 22Rv1 and C4-2 cells. **(E)** CCK-8 assay measuring cell viability in *HSPD1*-overexpressing versus control 22Rv1 and C4-2 cells at 24 h, 48 h, 72 h, and 96 h. **(F)** EdU proliferation assay (left: representative staining; right: quantitative analysis) in *HSPD1*-overexpressing versus control 22Rv1 and C4-2 cells. **(G)** Wound healing assay (top) and migration area quantification (bottom) in *HSPD1*-overexpressing versus control 22Rv1 and C4-2 cells. **(H)** Transwell migration/invasion assays with semiquantitative analysis (top: representative images; bottom: statistical plots) in *HSPD1*-overexpressing versus control 22Rv1 cells. **(I)** Excised subcutaneous 22Rv1 xenograft tumors from nude mice (OE-NC vs. OE-*HSPD1*, n = 5). **(J)** Longitudinal monitoring of tumor volume in 22Rv1 xenograft models. **(K)** Terminal tumor weight comparison between control and experimental groups. Statistical analysis is performed using two-sided t test **(A), (C), (D), (F), (G), (H), (K)** and tow-way ANOVA in **(E), (J);** Means ± SD, *P < 0.05; **P < 0.01; ***P < 0.001.

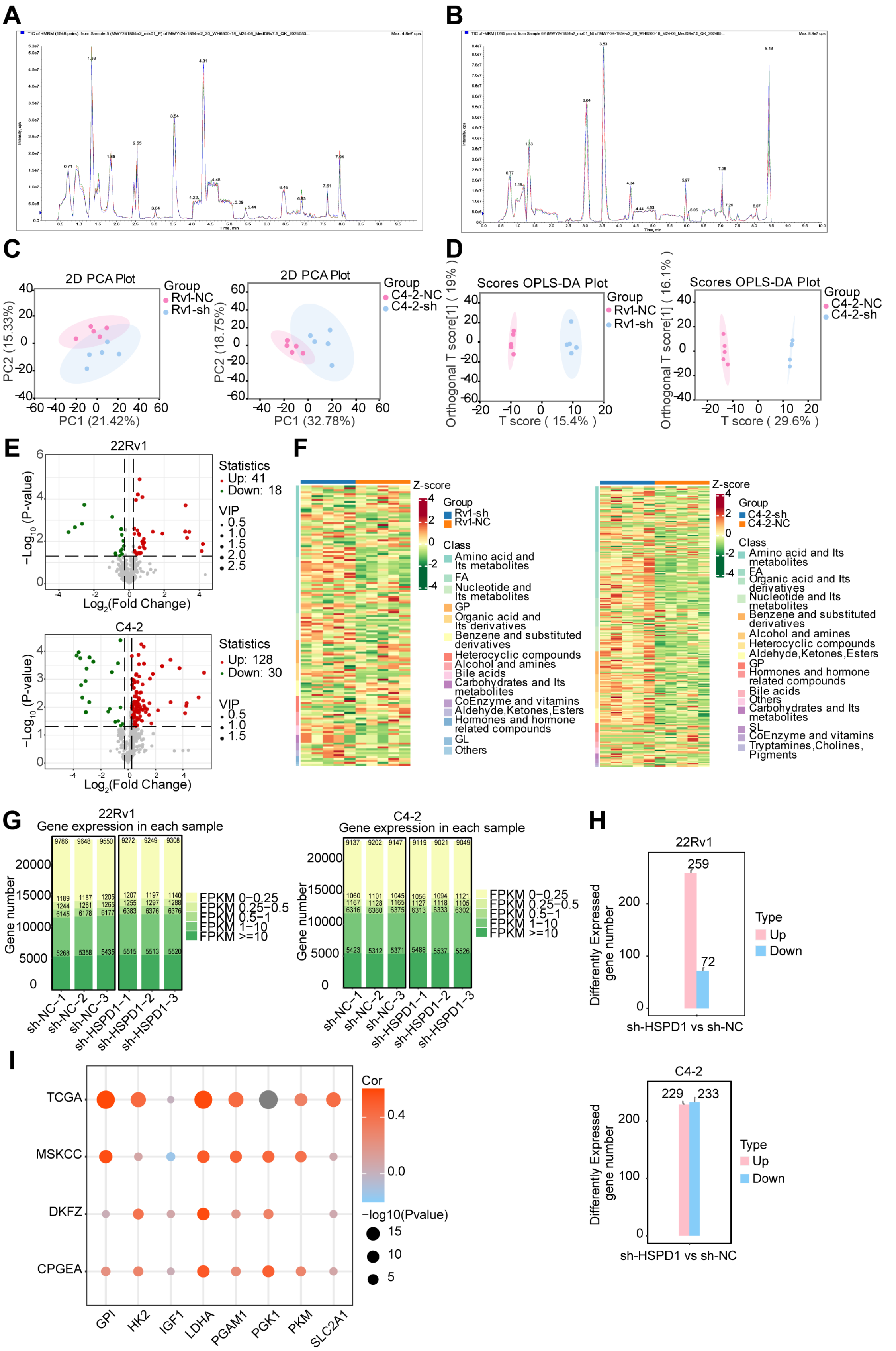

Fig. S4 Multi-omics profiling reveals molecular alterations upon *HSPD1* knockdown in 22Rv1 and C4-2 cells

(A) Overlaid total ion chromatogram (TIC) plots of QC samples acquired in positive ion mode by mass spectrometry. **(B)** Overlaid TIC plots of QC samples acquired in negative ion mode by mass spectrometry. **(C)** PCA plot of 22Rv1 and C4-2 cells in metabolomic analysis. **(D)** OPLS-DA plot of 22Rv1 and C4-2 cells in metabolomic analysis. **(E)** Volcano plot of differential metabolites in 22Rv1 and C4-2 cells (metabolomic dataset). **(F)** Hierarchical clustering heatmap of differential metabolites in 22Rv1 and C4-2 cells. **(G)** FPKM distribution of transcriptomic profiles in 22Rv1 and C4-2 cells. **(H)** Bar graph quantifying differentially expressed genes in 22Rv1 and C4-2 cells. **(I)** Coexpression bubble plot of *HSPD1* with glycolysis-related genes in TCGA, MSKCC, DKFZ, and CPGEA PCa cohorts.

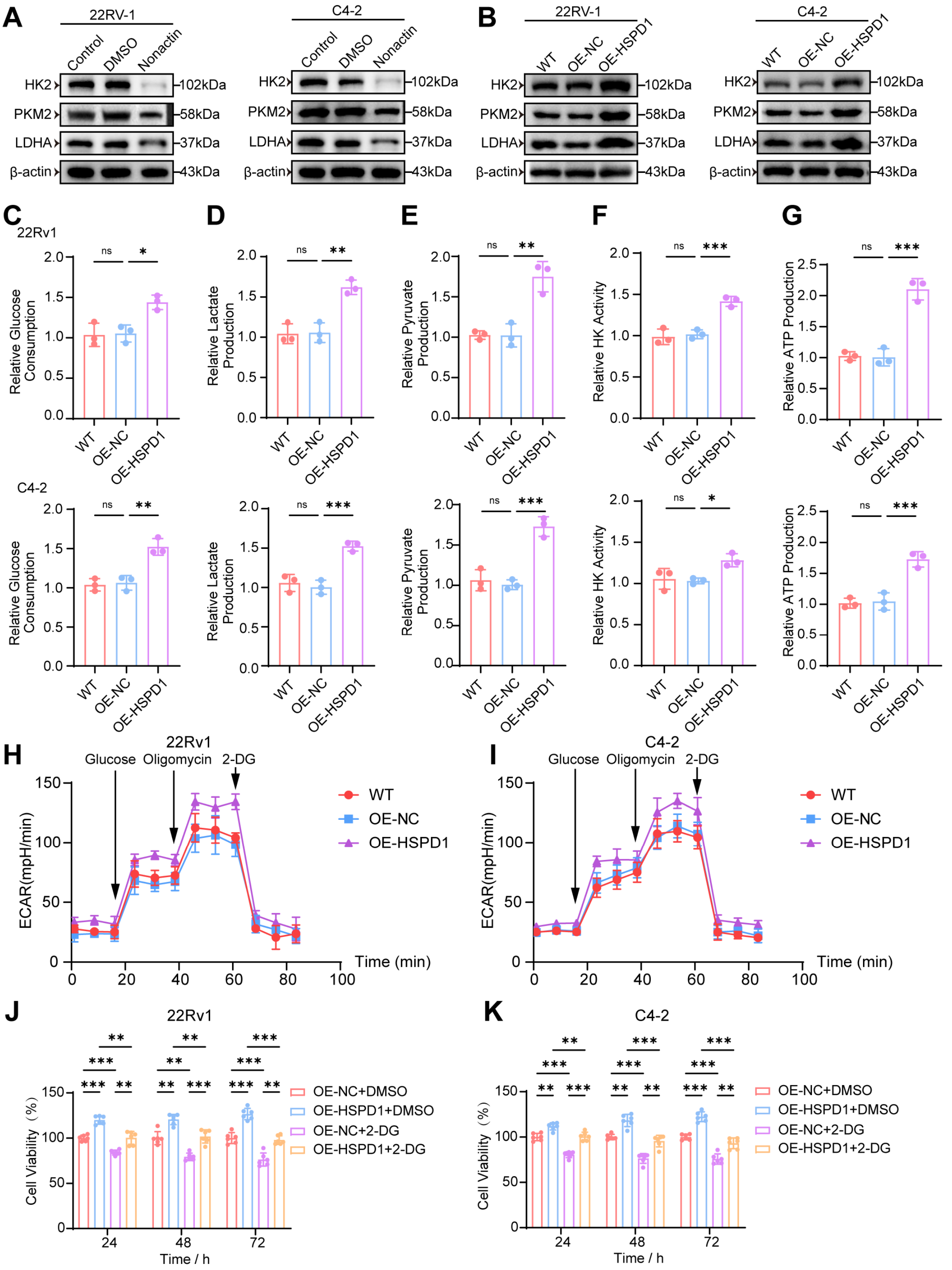

Fig. S5 *HSPD1* promotes the malignant progression of PCa cells by enhancing glycolysis

(A) Western blot of glycolytic enzymes in 22Rv1 and C4-2 cells following Nonactin intervention. **(B)** Western blot of glycolytic enzymes in 22Rv1 and C4-2 cells following

HSPD1 overexpression. **(C)** Measurement of relative glucose consumption in *HSPD1*-overexpressed 22Rv1 and C4-2 cells. **(D)** Measurement of relative lactate production in *HSPD1*- overexpressed 22Rv1 and C4-2 cells. **(E)** Measurement of relative pyruvate production in *HSPD1*- overexpressed 22Rv1 and C4-2 cells. **(F)** Measurement of relative HK activity in *HSPD1*- overexpressed 22Rv1 and C4-2 cells. **(G)** Measurement of relative ATP production in *HSPD1*-overexpressed 22Rv1 and C4-2 cells. **(H)** Measurement of ECAR in *HSPD1*- overexpressed 22Rv1 cells. **(I)** Measurement of ECAR in *HSPD1*- overexpressed C4-2 cells. **(J)** CCK-8 assay measuring cell viability in 22Rv1 cells across different experimental groups at 24 h, 48 h, and 72 h. **(K)** CCK-8 assay measuring cell viability in C4-2 cells across different experimental groups at 24 h, 48 h, and 72 h. Statistical analysis is performed using two-sided t test **(C), (D), (E), (F), (G), (J), (K);** Means ± SD, $*P < 0.05$; $**P < 0.01$; $***P < 0.001$.

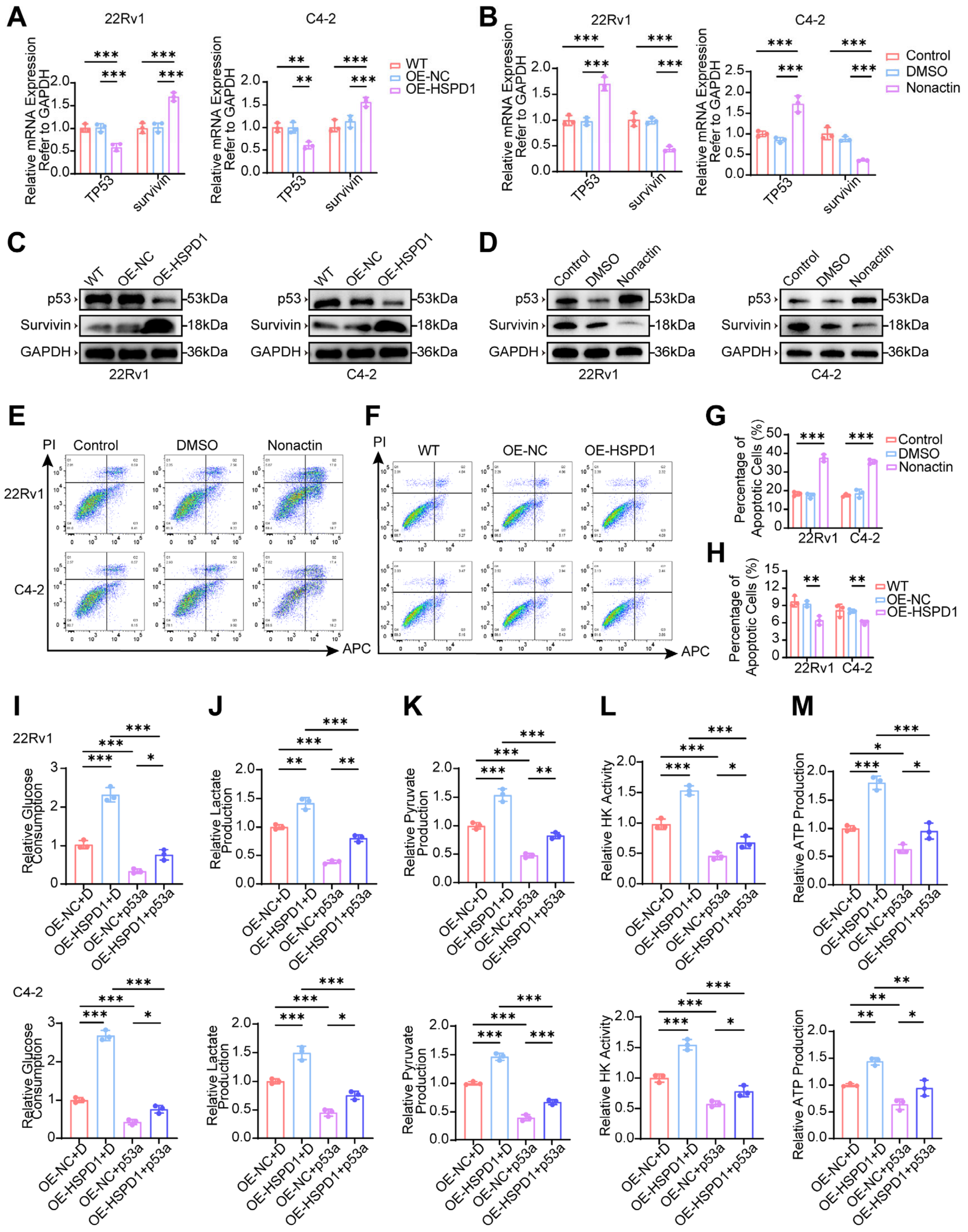

Fig. S6 HSP60 sustains PCa glycolysis by suppressing p53-mediated apoptosis

(A) qPCR quantification of TP53 and survivin (BIRC5) mRNA levels in *HSPD1*-overexpression 22Rv1 and C4-2 cells. **(B)** qPCR quantification of TP53 and survivin (BIRC5) mRNA levels in Nonactin-treated versus control 22Rv1 and C4-2 cells. **(C)**

Western blot analysis of p53 and Survivin protein expression in *HSPD1*-overexpression 22Rv1 and C4-2 cells. **(D)** Western blot analysis of p53 and Survivin protein expression in *HSPD1*- overexpression 22Rv1 and C4-2 cells. **(E)** Flow cytometry apoptosis assay in Nonactin-treated versus control 22Rv1 and C4-2 cells. **(F)** Flow cytometry apoptosis assay in *HSPD1*- overexpression 22Rv1 and C4-2 cells. **(G-H)** Quantitative comparison of apoptotic rates among different groups. **(I)** p53 activator-mediated change of glucose consumption in *HSPD1*-overexpression 22Rv1 and C4-2 cells. **(J)** p53 activator-mediated change of lactate production in *HSPD1*-overexpression 22Rv1 and C4-2 cells. **(K)** p53 activator-mediated change of pyruvate production in *HSPD1*-overexpression 22Rv1 and C4-2 cells. **(L)** p53 activator-mediated change of HK activity in *HSPD1*-overexpression 22Rv1 and C4-2 cells. **(M)** p53 activator-mediated change of ATP production in *HSPD1*-overexpression 22Rv1 and C4-2 cells. Statistical analysis is performed using two-sided t test **(A), (B), (G), (H), (I), (J), (K), (L), (M);** Means ± SD, $*P < 0.05$; $**P < 0.01$; $***P < 0.001$.

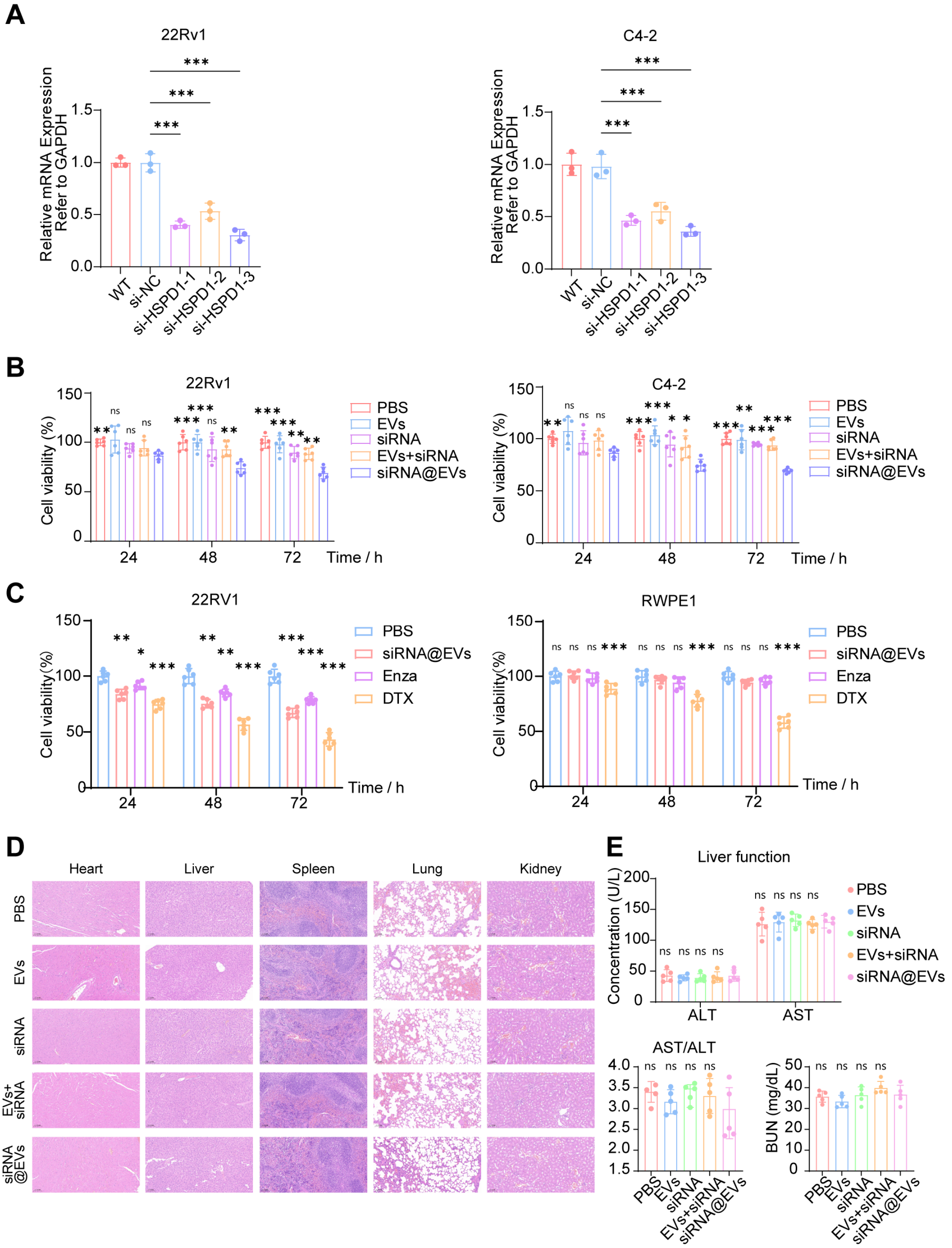

Fig. S7 Validation of *HSPD1* knockdown efficiency, cell viability assessment, and in vivo safety profiling

(A) qPCR analysis of *HSPD1* mRNA levels in 22Rv1 and C4-2 cells infected with si-*HSPD1*. **(B)** CCK-8 viability assessment of 22Rv1 and C4-2 cells under different

treatment conditions at 24 h, 48 h, and 72 h. **(C)** CCK-8 viability assessment of 22Rv1 and C4-2 cells under different existing treatments (siRNA@EVs, enzalutamide, docetaxel) at 24 h, 48 h, and 72 h. **(D)** Representative H&E-stained sections of major organs (heart, liver, spleen, lung, kidney) from nude mice across different experimental groups. **(E)** Detection of serum biochemical parameters in nude mice across different experimental groups. Statistical analysis is performed using two-sided t test **(A), (B), (C), (E);** Means ± SEM, *$P < 0.05$; **$P < 0.01$; ***$P < 0.001$.

Supplementary Tables

Supplementary Table 1. Characteristics of patients in Tongji PCa cohort

Sample lD	Age (year)	Gleason	T	N
1	59	6	T2c	N0
2	74	8	T3a	N0
3	57	6	T2a	NX
4	52	7	T3a	N0
5	58	7	T4	N1
6	62	7	T3b	NX
7	67	8	T3b	N0
8	68	7	T2	N1
9	67	9	T4	N1
10	69	7	T2c	NX
11	55	7	T2c	N0
12	74	9	T3b	N0
13	70	7	T2	N0
14	62	7	T2b	N1
15	72	7	T2a	N0
16	62	7	T3b	NX
17	55	7	T2c	N0
18	62	7	T2b	N0
19	51	6	T2a	N0

20	59	8	T2	N0
21	56	7	T3a	NX
22	65	8	T4	NX
23	67	8	T3a	N1
24	70	9	T2c	N0
25	66	8	T3b	NX
26	52	7	T2a	NX
27	64	7	T2	N0
28	71	7	T2a	NX
29	68	7	T2c	NX
30	65	9	T3b	N1
31	73	7	T2a	NX
32	59	6	T2a	N0
33	67	6	T2a	NX
34	71	7	T4	N1
35	61	7	T4	N0
36	73	7	T2b	N0
37	57	6	T3a	N0
38	60	7	T3a	N0
39	68	7	T4	N1
40	57	9	T4	N0
41	73	7	T4	N0

42	59	8	T4	N0
43	78	7	T2c	N0
44	75	9	T2a	NX
45	54	7	T4	N1
46	62	8	T4	N1
47	62	6	T2b	NX
48	51	8	T3b	N1
49	67	9	T4	N1
50	70	7	T2b	N0
51	76	6	T2a	NX
52	63	9	T2b	N0
53	81	9	T4	N1
54	56	7	T3a	NX
55	59	7	T2	N0
56	66	8	T3b	NX
57	64	8	T4	N1
58	70	8	T2a	NX
59	65	8	T3b	N1
60	72	8	T4	N1
61	68	7	T3b	NX
62	67	9	T4	NX
63	71	6	T2a	N0

64	74	8	T3b	Nx
65	72	9	T3a	N1
66	62	7	T2a	NX
67	59	7	T2b	N0
68	75	8	T2c	N0
69	66	7	T3b	N0
70	76	8	T3b	N0
71	67	9	T4	N1
72	74	9	T3b	NX
73	67	9	T3b	N0
74	64	9	T4	N1
75	67	8	T3b	N0
76	66	6	T2a	N0
77	66	7	T2	N0
78	58	7	T3b	N1
79	56	9	T4	N1
80	63	7	T2a	N0
81	67	6	T3a	NX
82	77	8	T3b	Nx
83	57	7	T2a	NX
84	77	9	T3a	N0
85	62	9	T3a	NX

86	73	8	T4	NX
87	59	7	T3a	N0
88	60	8	T4	N1
89	70	7	T2b	N0
90	60	6	T2	N0
91	60	7	T3b	NX
92	53	7	T2a	NX
93	65	7	T3a	NX
94	75	9	T4	NX
95	77	8	T4	N0
96	79	8	T2a	NX

Supplementary Table 2. Primer Sequences

Gene	Primer Type	Sequences
GAPDH	Forward	CTGGGCTACACTGAGCACC
	Reverse	AAGTGGTCGTTGAGGGCAATG
HSPD1	Forward	GCCAATGCTCACCGTAAGCCTTT
	Reverse	TGCCACAACCTGAAGACCAACCT
TP53	Forward	CAGCACATGACGGAGGTTGT
	Reverse	TCATCCAAATACTCCACACGC
BIRC5 (survivin)	Forward	AGGACCACCGCATCTCTACAT
	Reverse	AAGTCTGGCTCGTTCTCAGTG
HK2	Forward	GAGCCACCACTCACCCTACT
	Reverse	CCAGGCATTCGGCAATGTG
LDHA	Forward	ATGGCAACTCTAAAGGATCAGC
	Reverse	CCAACCCCAACAACTGTAATCT
PKM	Forward	ATGTCGAAGCCCCATAGTGAA
	Reverse	TGGGTGGTGAATCAATGTCCA
SLC2A1	Forward	GGCCAAGAGTGTGCTAAAGAA
	Reverse	ACAGCGTTGATGCCAGACAG
GPI	Forward	CAAGGACCGCTTCAACCACTT
	Reverse	CCAGGATGGGTGTGTTTGACC
PGAM1	Forward	GTGCAGAAGAGAGCGATCCG
	Reverse	CGGTTAGACCCCCATAGTGC

PGK1	Forward	TGGACGTTAAAGGGAAGCGG
	Reverse	GCTCATAAGGACTACCGACTTGG
IGF1	Forward	GCTCTTCAGTTCGTGTGTGGA
	Reverse	GCCTCCTTAGATCACAGCTCC

Supplementary Table 3. Related antibodies and their manufacturers

Antibody	**manufacturer**
HSP60 (D6F1) XP® Rabbit mAb	Cell Signaling Technology
GAPDH (14C10) Rabbit mAb	Cell Signaling Technology
β-Actin Rabbit mAb	ABclonal
[KO Validated] p53 Rabbit mAb	ABclonal
Survivin Rabbit mAb	ABclonal
Hexokinase II Rabbit mAb	Abcam
LDHA Rabbit pAb	ABclonal
PKM2-specific Rabbit mAb	ABclonal
HRP-conjugated Goat anti-Mouse IgG (H+L)	ABclonal
HRP-conjugated Goat anti-Rabbit IgG (H+L)	ABclonal

Supplementary Table 4. The sequences of all shRNA and siRNA oligonucleotides

sh-RNA/si-RNA	Sense	Antisense
sh-NC	GATCTGTTCTCCGAACGTGTCACGTTTCAAGAGAACGTGACACGTTCGGAGAATTTTTTC	AATTGAAAAAATTCTCCGAACGTGTCACGTTCTCTTGAAACGTGACACGTTCGGAGAACA
sh-*HSPD1*-1	GATCCGTGTTCAAGATGTTGCCAATAACTCGAGTTATTGGCAACATCTTGAACATTTTTT	AATTAAAAAATGTTCAAGATGTTGCCAATAACTCGAGTTATTGGCAACATCTTGAACACG
sh-*HSPD1*-2	GATCCGCTCCAGGGTTTGGTGACAATACTCGAGTATTGTCACCAAACCCTGGAGTTTTTT	AATTAAAAAACTCCAGGGTTTGGTGACAATACTCGAGTATTGTCACCAAACCCTGGAGCG
sh-*HSPD1*-3	GATCCAGACGATGCCATGCTCTTAAACTCGAGTTTAAGAGCATGGCATCGTCTTTTTTT	AATTAAAAAAAGACGATGCCATGCTCTTAAACTCGAGTTTAAGAGCATGGCATCGTCTG
si-NC	UUCUCCGAACGUGUCACGUTT	ACGUGACACGUUCGGAGAATT
si-*HSPD1*-1	CCUGCUCUUGAAAUUGCCATT	UGGAAUUUCAAGAGCAGGTT

si-*HSPD1*-2	GAGACAAAGAAAUUGGCAATT	UUGCCAAUUUCUUUGUCUCTT
si-*HSPD1*-3	AGACGAUGCCAUGCUCUUAAATT	UUUAAGAGCAUGGCAUCGUCUTT
